# Bacterial community structure transformed after thermophilically composting human waste in Haiti

**DOI:** 10.1371/journal.pone.0177626

**Published:** 2017-06-01

**Authors:** Yvette M. Piceno, Gabrielle Pecora-Black, Sasha Kramer, Monika Roy, Francine C. Reid, Eric A. Dubinsky, Gary L. Andersen

**Affiliations:** 1 Ecology Department, Earth and Environmental Sciences Area, Lawrence Berkeley National Laboratory, Berkeley, CA, United States of America; 2 Agricultural & Environmental Chemistry Graduate Group, University of California, Davis, CA, United States of America; 3 Sustainable Organic Integrated Livelihoods, Port-au-Prince, Haiti; Kyungpook National University, REPUBLIC OF KOREA

## Abstract

Recycling human waste for beneficial use has been practiced for millennia. Aerobic (thermophilic) composting of sewage sludge has been shown to reduce populations of opportunistically pathogenic bacteria and to inactivate both *Ascaris* eggs and culturable *Escherichia coli* in raw waste, but there is still a question about the fate of most fecal bacteria when raw material is composted directly. This study undertook a comprehensive microbial community analysis of composting material at various stages collected over 6 months at two composting facilities in Haiti. The fecal microbiota signal was monitored using a high-density DNA microarray (PhyloChip). Thermophilic composting altered the bacterial community structure of the starting material. Typical fecal bacteria classified in the following groups were present in at least half the starting material samples, yet were reduced below detection in finished compost: *Prevotella* and Erysipelotrichaceae (100% reduction of initial presence), Ruminococcaceae (98–99%), Lachnospiraceae (83–94%, primarily unclassified taxa remained), *Escherichia* and *Shigella* (100%). Opportunistic pathogens were reduced below the level of detection in the final product with the exception of *Clostridium tetani*, which could have survived in a spore state or been reintroduced late in the outdoor maturation process. Conversely, thermotolerant or thermophilic Actinomycetes and Firmicutes (e.g., *Thermobifida*, *Bacillus*, *Geobacillus*) typically found in compost increased substantially during the thermophilic stage. This community DNA-based assessment of the fate of human fecal microbiota during thermophilic composting will help optimize this process as a sanitation solution in areas where infrastructure and resources are limited.

## Introduction

Recycling human waste for beneficial use has been practiced since ancient times [1], but concerns related to disease transmission and how to handle high-density urban living situations brought about the use of infrastructure-intensive wastewater treatment plants throughout the industrialized world, requiring copious amounts of water for handling human waste. Unfortunately, some regions of the world remain limited in terms of water availability or infrastructure. For example, Haiti, even prior to the massive earthquake in 2010 that devastated much of its capital city, had no sewer infrastructure and no waste treatment. Many people were left without homes after the earthquake, and concerns about sanitation and the potential for disease outbreaks in the tropical environment were prominent. One organization that was positioned to help was Sustainable Organic Integrated Livelihoods (SOIL), a research and development nonprofit focused on recycling the nutrients in human waste and returning them to the soil through the process of thermophilic composting [2]. SOIL has collected and composted human waste for over 20,000 Haitians in Port-au-Prince and Cap-Haitien since 2006.

Despite the benefits of treating raw human waste via thermophilic composting, such as negligible water and infrastructure use and nutrient recycling, little is known about the dynamics of the microbial community throughout the process. Prior work has focused mainly on sewage sludge [3–5], which is already partially transformed by microbial processes prior to composting, or ambient-temperature composting toilets [6,7]. For example, Tønner-Klank and colleagues [8] studied composting toilet systems in Denmark and Sweden with fecal material in the study ranging in age from one month to many years, but the systems either did not reach thermophilic temperatures or did so for only a few days. Additionally, culturable *Escherichia coli*, *Enterococcus*, and *Salmonella* tests are widely used as end-point indicators for sewage treatment, yet because these taxa are a small fraction of the human gut microbiota [9] and can differ in longevity outside the gut, they give a limited perspective of the overall fate of human gut bacteria and potential pathogens during the composting process. Many of the numerically dominant gut bacteria are strict anaerobes (e.g., *Bacteroides*, various Ruminococcaceae and Lachnospiraceae) and therefore are not evaluated using the standard fecal indicator bacteria culture conditions, which are primarily aerobic. Of further consideration for warm climates, some bacteria, such as *E*. *coli* (and *Salmonella*, to a lesser extent), can survive in tropical environments outside the human gut [10,11], leading to an over-estimation of gut-bacteria survival in some cases. *E*. *coli* also has been shown to be part of the soil microbiota, especially in (sub)tropical soils [12,13]. The Enterococci are known to be hardy [14] and so their presence, too, is not strictly indicative of recent human waste exposure. Both Pourcher and colleagues [4] and Tønner-Klank and colleagues [8] have noted limitations related to using culture-based fecal indicator bacteria methods to evaluate the hygienic status of compost. A more comprehensive test to evaluate how the fecal-associated microbial community structure changes during thermophilic composting would provide valuable information about this method of treating human waste.

Modern molecular methods can track large numbers of organisms in complex sample matrices over time or throughout a process. In the last couple decades, several methods have been applied specifically to compost, such as clone library analysis of 16S rRNA genes [15,16], denaturing gradient gel electrophoresis [3,17–21], single-strand-conformation-polymorphism [22], (Automated) Ribosomal Intergenic Spacer Analysis **(**ARISA) [23], membrane lipid analyses [21], a medium-resolution compost-targeted DNA microarray (COMPOCHIP) [5,24], 16S rRNA gene iTag sequencing [25], and recently metagenomic analysis [26]. None though, have been used specifically to track the fate of human-associated bacteria from primary human waste through the thermophilic composting process. PhyloChip analysis uses sets of DNA probes fixed on a glass surface to which labeled sample DNA is hybridized [27,28] in order to examine differences in relative abundances of bacteria (or archaea) among samples. This analytical tool allows a complex microbial community analysis to be conducted rapidly in a single test using genomic DNA extracts as starting material. The PhyloChip is capable of detecting taxa in extremely low abundance (less than 0.05 ng (<0.5 pM) amplicon [28] or 10^-4 of total abundance [27,29], and is capable of distinguishing nearly 60,000 taxa [28], making complex microbial interactions traceable, in this case focused on the presence and relative abundances of human gut bacteria and opportunistic pathogens throughout the thermophilic composting process. The PhyloChip microarray has been used previously with human stool samples and with fecal-associated environmental samples [30–34] including samples used for fecal source tracking [35,36].

Herein we describe a bacterial community analysis that was conducted over 6 months at two SOIL composting facilities located in Haiti. Samples were taken from various stages of the composting process, beginning with fresh waste collected at a variety of public and household toilets and ending with finished (bagged) commercially available compost. Using this analysis, we observed changes in the bacterial community structure reflecting a transformation from typical fecal microbiota to typical compost microbiota through thermophilic composting and an extended curing phase.

## Materials and methods

### Sampling locations

Two of SOIL’s composting facilities were sampled in Haiti: Port-au-Prince (PauP; 18°36'44.40"N, 72°20'17.78"W) and Cap-Haitien (Cap-H; 19°40'38.20"N, 72°06'49.07"W) at various times during Fall 2013 through Summer 2014. The field collections did not require permits or permissions; they are part of the regular working procedures for SOIL. This research did not involve endangered or protected species. Human waste from the Ecosan toilets was retrieved from homes and emergency shelters within 20 km of each facility.

Each facility collected waste from public and private SOIL-serviced toilets. Material from the toilets in PauP consisted of a mix of feces and small fibers of sugarcane bagasse. The mixture was typically collected in approximately 20 L or 60 L containers for up to two weeks prior to being transported to the composting site where it was added to 12 cubic m, three-walled composting bins built from shipping pallets stuffed with large pieces of sugarcane bagasse, all sitting on layers of sugarcane bagasse on compacted soil, as detailed by Berendes et al. [37]. The piles were designed for leachate to flow into planted swales between the bins. When constructing the piles, the buckets were emptied into the bins, bagasse was added in layers, and bins were thusly filled until the waste was level with the top of a bin. Piles were then covered with a thick layer of bagasse to protect them from wind and insect vectors and to maintain heat. Occasionally urine collected from the urine-diverting toilets was added to the piles to increase moisture content.

The piles remained open to the air throughout the process and in PauP were turned monthly for four months. Turning the piles periodically was initiated after Berendes and colleagues, measuring temperatures in piles constructed the same way as described at the PauP SOIL facility during an earlier study [37], discovered compost bin corners had lower temperatures than recommended for inactivating pathogens (e.g., *Ascaris* ova). A 2003 EPA report [38] states temperatures of 55°C for 3 days (or 40°C for 5 days with 4 hours at 55°C) is recommended for pathogen reduction to acceptable levels (<1 viable helminth ova per 4 g solids). Pecson et al. [39] demonstrated rapid inactivation of *Ascaris* eggs at pH 7 and 50°C (<2 h) and moderately fast inactivation at 40°C (12 days), but eggs were not inactivated for several months at 30°C. Corner temperatures of SOIL compost piles, as reported by Berendes et al., ranged from ~35–51°C between roughly Day 3–120. Due to the low corner temperatures, the composting process at SOIL facilities was modified to include a monthly redistribution of the compost material by turning to assure all material reached at least 55°C for at least several days, and it is typically much longer. The thermophilic stage generally lasts from three to four months in the middle of the piles. The temperatures at the center of a pile reported by Berendes and colleagues ranged from 60–70°C during the first two weeks of composting and then remained at or above 58°C until moved to a windrow. While compost pile temperatures are no longer monitored continuously at SOIL facilities and so are not available for the samples reported here, an example temperature profile for composting piles at SOIL’s PauP facility spanning six weeks during 2014 is provided in S1 Fig (courtesy of SOIL). Material for the current study was then moved from bins to windrows to complete the composting process. The windrow piles were considered curing from four to nine months. The composting process is considered finished and material ready for distribution when temperatures drop to ambient–generally six to nine months post pile initiation.

The composting facility at Cap-H was similar to that at PauP except that crushed peanut shells were used in addition to bagasse as cover material in the toilets (however bagasse was still used exclusively for bulking material at the compost site) and the four-walled bins were situated on concrete slabs. Additionally, the leachate draining from the bottom of the piles was collected and reintroduced to the top of each pile. The Cap-H piles emitted a strong ammonia odor, whereas there was no such odor observed from the PauP piles. The Cap-H piles were turned initially after 2.5 months and then again every two weeks for 12 weeks before being moved to windrows with curing times similar to PauP.

### Sample collection

Samples were collected from various sources at one of four composting stages: buckets retrieved from the field (Bucket), thermophilic compost piles (Thermo), curing compost in windrows (Curing), or bagged compost (Bagged). Bucket samples ranged in age from 1 to 14 days old at the time of sampling. Buckets were collected weekly or twice per month, depending on toilet type, from various locations around either city. Thermophilic compost piles comprised those with temperatures ranging from 29°C to 78°C (based on periodic measurements made by SOIL personnel, and depending on the location within the pile; centers are hottest) and were in the range of 30–45 days post pile closure. Curing compost piles had temperatures ranging from 21°C to 55°C (also based on typical measurements and depending on the location within the pile), and samples were collected from piles ranging in age from roughly six to seven months. Bagged compost, age of at least one year post pile closure, was sampled from three different bags of finished compost material that was ready to be distributed to the community for use.

Compost samples were collected from 50 cm below the surface of a pile. A shovel was used to dig to the desired depth, and compost was collected with a sterilized hand trowel. Samples were taken from three points in each pile, two at the edges and one in the center to obtain an adequate representation of the pile, though turning the piles ensured all material had a chance to be at the center (the hottest part of a pile) during part of the process.

### Field DNA extraction

Approximately five-gram subsamples were homogenized using 15mL tubes containing stainless steel beads by vortexing on a Vortex-Genie 2 Shaker (Scientific Industries, Bohemia, NY) at 2700 rpm for five minutes. 0.2g of homogenized material was extracted using ZR Fecal DNA MiniPrep kits (Zymo Research, Irvine, CA). Tubes were affixed to a vortex and cells lysed using the highest vortex setting for 5 min. Particulates were pelleted using a table-top centrifuge (Galaxy MiniStar, VWR, Radnor, PA) at max speed (2,000xg) for 5 min. Extractions were carried out using a ZR Fecal DNA kit (Zymo Research Corp., Irvine, CA) and modified extraction protocol, which is presented in detail in [40]. Extracted genomic DNA (gDNA) was shipped to LBNL (Berkeley, CA) for quantification using Qubit dsDNA HS Assay Kit (Life Technologies, Grand Island, NY) and processing for PhyloChip G3 analysis.

SOIL employees, who underwent a three-day DNA extraction process training course in Haiti, performed all sample collections and extractions. The individuals were taught basic sample handling principles as well as the aforementioned DNA extraction protocol. Each individual was required to pass an exam upon completion of the course prior to performing extractions on samples within the data set.

Genomic DNA (gDNA) was shipped overnight via DHL from Port-au-Prince, Haiti to Berkeley, CA. Samples were placed in a 12 in^3^ Styrofoam cooler with six PolarPack Standard Refrigerant Gel Packs (Sonoco Protective Solutions, Hayward, CA). MonitorMark Time Temperature Indicators (3M, St. Paul, MN) were activated in each shipment to ensure the samples were not exposed to temperatures over 18°C.

### 16S rRNA gene amplification and PhyloChip processing

Genomic DNA (1 ng) was used to amplify 16S rRNA genes via polymerase chain reaction (PCR) using primers 27F (5’- AGAGTTTGATCCTGGCTCAG -3’) and 1492R (5’- GGTTACCTTGTTACGACTT -3’). PCR conditions were as described previously [28,32]. Each sample (1 ng template DNA) was amplified in four replicate 25 μL reactions, one at each annealing temperature (50, 52, 54, and 56°C) for 25 cycles. PCR products were pooled for each sample, purified using the Qiagen MinElute PCR kit (Qiagen, Valencia, CA), and recovered in 20 μL elution buffer. One microliter of each sample was quantified using gel (2% agarose) electrophoresis in comparison to standards.

Purified amplicons (500 ng) were applied to the G3 PhyloChip (Second Genome, South San Francisco, CA), an Affymetrix-platform microarray comprising16S rRNA gene sequences from bacteria and archaea. The G3 PhyloChip design and analysis is detailed in Hazen et al. ([28], Supp. Mat.). Samples were fragmented, labeled, and hybridized to the arrays and then probe fluorescence intensities were captured and processed for data analysis following [28].

### Data analysis

All arrays included a spike-mix with known amounts of non-16S rRNA genes (total 202 ng); probe fluorescence intensities of these positive controls were used to scale intensities for each sample. Bacterial operational taxonomic units (OTU) were called present in samples when they met the following criteria (based on descriptions in [28]): Stage1 pf 0.92, min_q1 0.8, min_q2 0.93, min_q3 0.98; post-Stage 2: min_q1 0.22, min_q2 0.40, min_q3 0.42. Additional criteria for this dataset were 1) an OTU had to be present in at least five (of 54) samples to be used for binary and relative intensity comparisons, and 2) a ‘human-associated’ subset of OTU comprising OTU present in at least 12 of the 24 ‘Bucket’ samples. Two thermophilic samples from PauP were excluded when calculating average OTU binary calls or intensities for the Thermo stage for comparison with other stages because the samples in question had very similar profiles to bucket samples, which may have been because part of that pile did not reach thermophilic conditions by the time of sampling. It is possible these two samples had been edge material prior to turning.

After establishing the entire study dataset, PauP and Cap-H samples were analyzed separately because the piles were constructed differently in the two locations, which may have influenced the development of the thermophilic composting community.

Many taxa were unclassified beyond family or genus and are referred to by the generic term ‘taxa’ in parts of the text. Binary data (presence/absence calls) were used to reflect relative richness, whereas probe intensity values (scaled to internal standards, standardized to the entire dataset, and square-root transformed) were used to assess relative abundance across samples. Differences in mean relative richness or relative abundance, typically for higher taxonomic levels, were tested using Kruskal-Wallis and Dunn tests with Benjamini–Hochberg correction in R [41,42], whereas contributions of finer-resolution taxa to Bray-Curtis similarities were examined using SIMPER (similarity percentages). SIMPER analysis has been used in many studies (e.g., [43–49]) following an overall test for differences among groups (e.g., ANOSIM) to identify variables contributing the most (and most consistently) to similarities within groups and dissimilarities between groups. This method was developed [50] to evaluate contributions from many variables to multivariate community structure because of the (likely) high correlation structure among those variables, which is unknowable when there are many more variables than samples with which to estimate each correlation parameter, thereby contra-indicating using traditional comparison methods. Non-metric Multi-Dimensional Scaling (NMDS) plots were generated and Analysis of Similarity (ANOSIM) and similarity percentage (SIMPER) analyses were conducted using Primer-E software [51]. SourceTracker analysis [52] was conducted in R using default parameters to track the fate of the initial untreated microbial community throughout the composting process. Binary data from untreated bucket samples were used as the SourceTracker training set, and samples from thermophilic, curing and bagged treatment stages were evaluated as sink samples to estimate the proportion of taxonomic richness derived from the untreated source. Data for pie charts were generated from 1070 OTU (Cap-H) or 784 OTU (PauP) having the greatest relative abundance differences across compost stages (12 paired stage comparisons, top 100 OTU per pair) or having at least a 10-fold change in average relative abundance across compost stages within each location (the list was de-replicated to remove OTU appearing twice because of being selected in multiple paired comparisons or by both methods). Subsequently, OTU classified within each species-level taxon were then grouped according to their trends throughout the composting process and intensity ranges; intensities for OTU so grouped were then averaged and presented in bar charts as supplemental tables.

The microarray data are available at http://greengenes.secondgenome.com/downloads/phylochip_datasets in LBL_MIAME_PhyloChip_Haiti-thermophilic-composting_Piceno_2017.zip.

## Results

### Fate of human-associated bacteria during thermophilic composting

A primary goal of this study was to assess the fate of human fecal bacteria through the process of thermophilic composting. Toward that end, we examined the data for bacteria associated with human fecal material defined in one of three ways: 1) bacteria in the Haitian source material present in at least half the Bucket samples, 2) bacteria considered human gut ‘common-core’ members (*sensu* Qin et al. [9]), and 3) all OTU associated with the Bucket samples (Source Tracker analysis). 1768 OTU were called present in at least 12 of the 24 Bucket samples (50% of the combined data from both locations); these were considered a stricter set of ‘human-fecal-associated’ bacteria than the full set of OTU in the starting material and were used for the first analysis. Although all buckets also contained bagasse or crushed peanut shells, the human fecal material is considered to have provided the preponderance of bacteria in Bucket samples, so this was deemed a good working criterion for human-associated communities for these samples. Multiple probes were assigned to each specific OTU, and stringent criteria to determine the presence or absence of any OTU resulted in a binary call (i.e., 0 for absent, 1 for present). Additional analysis examined the average fluorescent intensity of probes assigned to each OTU to assess the relative abundance differences from sample to sample. One OTU (# 24510) contained a sequence affiliated with *Subdoligranulum* in four samples by binary calls–and also had high intensity values in many samples–but the OTU did not make the initial cutoff for being called present in at least five samples (from the full set of 54 samples) and so was not included in the main dataset. Bacteria affiliated with this genus are quite common in human fecal material [9]. Additionally, the nearest named neighbor is *Faecalibacterium prausnitzii*, which is strictly anaerobic and non-spore-forming [53], and was present in greatest relative abundance in the Bucket samples.

**Fig 1:**
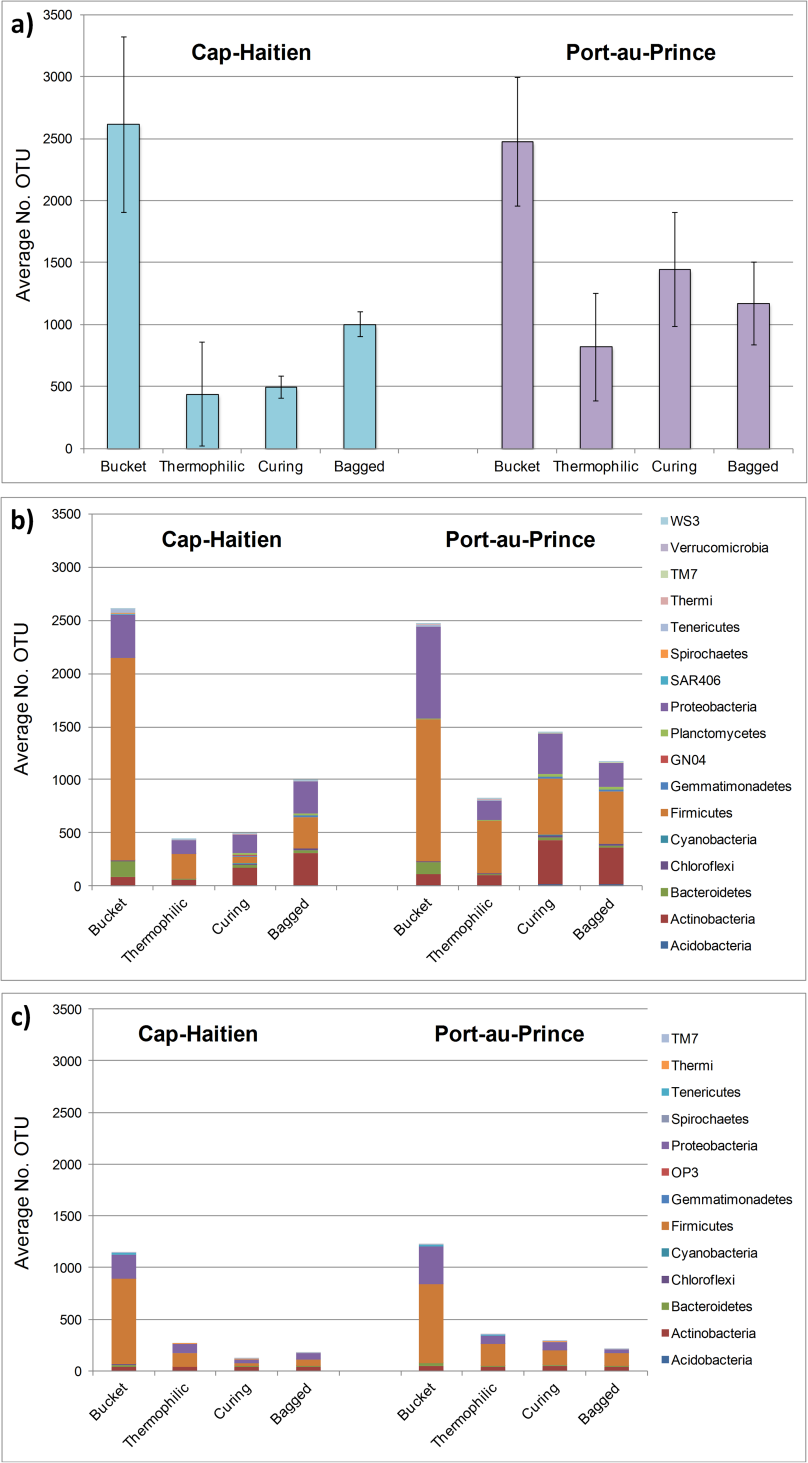
OTU relative richness calculated at each stage of a thermophilic composting process at two locations: Cap-Haitien and Port-au-Prince, Haiti. a) average number of OTU per composting process stage, error bars indicate standard deviation; b) distribution of OTU within phyla containing at least five OTU (main dataset of 7531 OTU); c) distribution of OTU within phyla comprising the 1768 OTU defined as human gut associated for this dataset (present in at least half the Bucket samples). Number of samples included per location: Bucket (n = 12), Thermophilic (n = 9 in Cap-Haitian, n = 7 in Port-au-Prince), Curing (n = 3), and Bagged (n = 3).

It was previously determined that the PhyloChip has a dynamic range of detection of over four orders of magnitude; that is, an rRNA gene copy present at 5:10,000 the concentration of the dominant rRNA sequence can still be identified [27,28]. This yields a greater detection sensitivity than amplicon sequencing performed at typical depths (Probst 2014). For most of the samples in this current study, the thermophilic composting process caused the fecal-associated bacteria to decline below this detection limit, with precipitous declines in relative richness and relative abundance at the thermophilic stage (Fig 1, S1 Table, S2 Table, S9 Table, S10 Table). A test for differences in passing number of OTU at each stage yielded overall p<0.001 for Cap-H and PauP (Kruskal-Wallis (KW)), and post-hoc tests (Dunn with Benjamini-Hochberg correction for multiple comparisons (BH)) showed the greatest differences occurred between Bucket and Thermo stages (p<0.0001 for Cap-H, p<0.001 for PauP). Specifically, both locations had significant differences in Proteobacteria (using alpha-adjusted p-values of <0.0029: Cap-H p = 0.0005, PauP p = 0.0003) and Bacteroidetes (Cap-H p = 0.0003, PauP p = 0.0004). Posthoc BH-corrected comparisons confirmed significant differences between Bucket and Thermo stages for both locations (S9 Table). Additionally, Cap-H had significant differences for Actinobacteria (p = 0.0016), Chloroflexi (p = 0.0002), Firmicutes (p = 0.0001), and Planctomycetes (p = 0.0008), Spirochaetes (p = 0.0002), Tenericutes (p = 0.0005), and other phyla, whereas most of these phyla had p>0.002 (but <0.005 for all but the last two) for PauP (S9 Table).

The binary data for the human-associated set of OTU (1768) revealed OTU classified in the following taxa were present in at least half the starting material samples yet were reduced below detection in finished compost: *Prevotella* and Erysipelotrichaceae (100% reduction of initial presence), Ruminococcaceae (98–99%), Lachnospiraceae (83–94%), *Escherichia* and *Shigella* (100%). Within the Ruminococcaceae, all OTU classified as *Ruminococcus*, *Oscillospira*, and *Clostridium* declined below detectable levels, and only 1 or 2 (Cap-H or PauP, respectively) remained of the original 528 OTU in *Faecalibacterium*. The remaining OTU were all unclassified. Within the Lachnospiraceae, *Blautia*, *Butyrivibrio*, *Eubacterium*, *Lachnobacterium*, *Lachnospira*, and *Ruminococcus* declined below detection. *Clostridium*, *Coprococcus*, and *Roseburia* had two or fewer OTU remaining, so the preponderance of the OTU within Lachnospiraceae remaining in the bagged samples also were unclassified.

There were large declines in Firmicutes and Proteobacteria OTU prevalence at the Thermo stage that continued through the Curing stage (Fig 1C, S9 Table (though the adjusted p-value was borderline for Firmicutes at PauP)). For example, from S1 Table and S2 Table we see various Lachnospiraceae decreased in relative abundance in the thermophilic stage (especially large decline at Cap-H) and then decreased again slightly at the Curing stage. The same tables show several bacteria within the phyla Bacteroidetes and Firmicutes had marked decreases at the Thermo stage: *Bacteroides*, *Parabacteroides*, *Prevotella*, *Rikenellaceae II*, *Lactobacillus ruminis*, an unclassified Catabacteriaceae, *Blautia*, *Ruminociccus obeum*, *Coprococcus*, *Eubacterium*, *Faecalibacterium*, *Oscillospira*, and a few *Clostridium*, most of which never recovered. Notably, one set of OTU classified as *Eubacterium rectale* (within the PhyloChip genus classification of *Roseburia*, so placed because of sequence similarity as opposed to the original sequence assignment when submitted) in both locations remained relatively constant, whereas other OTU classified within *Roseburia* declined to effectively no fluorescent hybridization signal. The differences in OTU responses may be attributable to probes in one OTU set being more or less specific than probes in a different set or due to real differences in populations within a genus/species. The number of Firmicutes OTU called present rebounded slightly in the finished compost at Cap-H (Fig 1C, S9 Table adjusted p-value = 0.127 Bucket:Bagged).

Within the Proteobacteria, relative abundances of the Enterobacteriales (*Escherichia*, *Klebsiella* (at Cap-H), *Shigella*) decreased substantially at both locations (S10 Table), and several of these members declined more than any other bacteria by the end of composting (Bagged samples) at PauP. This was similar to what occurred at Cap-H. Although *Enterococcus* OTU did not show sufficient changes in relative abundance to be included in S1 Table (Cap-H), there actually were greater reductions in relative intensities between Bucket and Thermo stages at Cap-H than at PauP (S4 Table; Kruskal-Wallis p<0.001 for Cap-H, p<0.01 for PauP). The change averaged for OTU in *E*. *casseliflavus* at Cap-H was -3513 fluorescence intensity (FI) units (BH-corrected post-hoc p<0.001) and at PauP it was -935 (p = 0.195). For *E*. *faecalis* it was -3907 FI units at Cap-H (p<0.001) versus -727 at PauP (p = 0.21). For unclassified OTU within *Enterococcus*, it was -4094 FI units at Cap-H (p<0.001) and -1301 at PauP (p = 0.21).

Within Tenericutes (specifically the Erysipelotrichales), there were multiple sets of *Eubacterium biforme* OTU based on their responses throughout the composting process (S1 Table and S2 Table). One set of OTU declined substantially at the Thermo stage and was essentially absent in the latter two stages at both locations; the other sets declined moderately at the Thermo stage and remained roughly the same throughout. The OTU classified as *Bulleidia* followed the same course as the *E*. *biforme* set that declined below detection (Cap-H and PauP showed statistical differences across compost stages (S10 Table). Within the Phylum Verrucomicrobia, average OTU abundance for *Akkermansia* appeared to decline, but there were not statistically significant differences once adjusting for multiple comparisons (Cap-H p = 0.009, PauP p = 0.054).

A representative of *Turicibacter* declined slightly in the Curing samples, but increased again in the Bagged samples (S6 Table). While this genus is not mentioned by Qin et al. [9] as part of the common core of gut bacteria, it has been found in human and other animal fecal samples [54] and was isolated originally from the blood of a patient with appendicitis [55].

For the second analysis, the human gut ‘common-core’ bacteria were those defined in Supplementary Table 8 of Qin et al.’s study [9]. S3 Table (this study) shows the percentage of samples at each location with OTU classified within common core taxa or the nearest representatives within the genus. *Blautia producta* was found in the Haitian samples as opposed to *B*. *hansenii* listed in Qin et al.’s Table 8, but these two species are closely related [56]. Furthermore, *R*. *obeum*, part of the ‘common core’ gut bacteria [9] was reclassified recently as *Blautia obeum* [57], so PhyloChip OTU affiliated with previously-classified *R*. *obeum* sequences are herein classified within *Blautia*. The data in Table 1 and S3 Table demonstrate the Bucket material encompassed typical fecal bacteria, containing members of the same genus or species as the ten most prevalent named bacterial taxa in the human gut according to metagenome data [9]: *Faecalibacterium prausnitzii*, *Roseburia* (a sequence similar to the Qin et al. *R*. *intestinalis* sequence is included in PhyloChip OTU 18119 *Roseburia* sp_unclassified), Dorea (formicigenerans in Qin et al. vs. sp_unclassified in Haitian samples), *Bacteroides* of various spp. (e.g., *vulgatus* sequences are found in OTU 68563 and 68486 (both are assigned to sp_unclassified in PhyloChip taxonomy), *uniformis* (not specifically identified in the Haitian samples, but a few other named sp. were detected, as well as sp_unclassified)), *Clostridium* sp. SS2-1 (PhyloChip OTU 20595), *Eubacterium* (*E*. *hallii* in Qin et al. vs. sp_unclassified in Haitian samples), *Coprococcus* (*C*. *comes* in Qin et al. vs. *eutactus* and sp_unclassified in Haitian samples), and *Eubacterium rectale* (classified within Genus Roseburia in PhyloChip taxonomy). There were several *Bacteroides* spp. missing from the Bucket samples, but most of the rest of the common-core bacteria were well-represented. Table 1 presents the subset of Qin and colleagues’ ‘common core’ gut bacteria [9] that were present at any composting stage and shows a decline to below detection for almost all taxa through the thermophilic composting process. As with the fecal-associated bacteria set of OTU, the reductions were more rapid at Cap-H than at PauP and remained lower more often at the end of the process.

**Table 1:** Average percentage of samples at each stage with human gut microbiome ‘common core’ bacteria called present in the Haitian waste (source) material or compost samples.

				Cap-Haitien				Port-au-Prince			
Class	Family	Genus or Species	# OTU averaged	Bucket (n = 12)	Thermo (n = 9)	Cured (n = 3)	Bagged (n = 3)	Bucket (n = 12)	Thermo (n = 9)	Cured (n = 3)	Bagged (n = 3)
Actinobacteria	Bifidobacteriaceae	Bifidobacterium adolescentis	2	25 (*3*.*0+/-0*)	0	0	0	8 (*1*.*0+/-0*)	22 (*2*.*0+/-0*)	0	0
		Bifidobacterium pseudocatenulatum	1	0	0	0	0	17 (*2*)	33 (*3*)	0	0
		Bifidobacterium (unclassified)	3	22 (*2*.*7+/-2*.*5*)	7 (*0*.*7+/-0*.*6*)	0	0	19 (*2*.*3+/-2*.*1*)	30 (*2*.*7+/-1*.*5*)	0	0
	Coriobacteriaceae	Collinsella (unclassified)	2	0	39 (*3*.*5+/-0*.*7*)	0	33 (*1*.*0+/-1*.*4*)	8 (*1*.*0+/-0*.*0*)	72 (*6*.*5+/-0*.*7*)	0	0
Bacteroidia	Bacteroidaceae	Bacteroides fragilis	2	33 (*4*.*0+/-0*)	0	0	0	8 (*1*.*0+/-0*)	0	0	0
		Bacteroides massiliensis	1	25 (*3*)	0	0	0	58 (*7*)	11 (*1*)	0	0
		Bacteroides ovatus	5	42 (*5*.*0+/-1*.*6*)	0	0	0	25 (*3*.*0+/-2*.*4*)	9 (*0*.*8+/-0*.*4*)	0	0
		Bacteroides plebeius	1	33 (4)	0	0	0	0	11 (1)	0	0
		Bacteroides (unclassified)	9	41 (*4*.*9+/-1*.*8*)	0	0	0	17 (*2*.*0+/-2*.*2*)	7 (*0*.*7+/-0*.*5*)	0	0
	Porphyromonadaceae	Parabacteroides distasonis	1	50 (*6*)	0	0	0	58 (*7*)	0	0	0
		Parabacteroides (unclassified)	2	25 (*3*.*0+/-1*.*4*)	0	0	0	17 (*2*.*0+/-1*.*4*)	0	0	0
	Prevotellaceae	Prevotella copri	14	42 (*5*.*1+/-2*.*3*)	0	0	0	26 (*3*.*1+/-1*.*5*)	10 (*0*.*9+/-0*.*4*)	0	0
Bacilli	Enterococcaceae	Enterococcus faecalis	9	35 (*4*.*2+/-2*.*0*)	0	15 (*0*.*4+/-0*.*5*)	0	31 (*3*.*8+/-0*.*4*)	19 (*1*.*7+/-1*.*2*)	0	0
	Streptococcaceae	Streptococcus (unclassified)	294	44 (*5*.*3+/-2*.*1*)	4 (*0*.*3+/-0*.*7*)	0.1 (*0*.*0+/-0*.*1*)	0.2 (*0*.*0+/-0*.*1*)	23 (*2*.*8+/-1*.*8*)	23 (*2*.*1+/-1*.*3*)	1 (*0*.*0+/-0*.*1*)	0.5 (*0*.*0+/-0*.*1*)
Clostridia	Clostridiaceae	Clostridium acetobutylicum	1	75 (*9*)	0	0	0	25 (*3*)	33 (*3*)	33 (*1*)	33 (*1*)
		Clostridium hiranonis	3	50 (*6*.*0+/-0*)	4 (*0*.*3+/-0*.*6*)	0	0	6 (*0*.*7+/-0*.*6*)	11 (*1*.*0+/-0*)	0	0
		Clostridium sordellii	1	8 (*1*)	0	0	0	8 (*1*)	22 (*2*)	33 (*1*)	0
		Clostridium tetani	1	8 (*1*)	0	0	0	25 (*3*)	11 (*1*)	0	67 (*2*)
		Clostridium (unclassified)	126	40 (*4*.*8+/-3*.*1*)	7 (*0*.*6+/-0*.*8*)	0.3 (*0*.*0+/-0*.*1*)	4 (*0*.*1+/-0*.*5*)	42 (*5*.*0+/-3*.*4*)	28 (*2*.*5+/-1*.*9*)	22 (*0*.*7+/-0*.*9*)	25 (*0*.*7+/-1*.*1*)
	Fm XI. Incertae Sedis	Clostridium ultunense	1	0	11 (*1*)	0	0	8 (*1*)	22 (*2*)	0	33 (*1*)
		Clostridium (unclassified)	3	0	0	0	22 (*0*.*7+/-0*.*6*)	8 (*1*.*0+/-0*.*0*)	22 (*2*.*0+/-0*.*0*)	33 (*1*.*0+/-0*.*0*)	56 (*1*.*7+/-0*.*6*)
	Fm XIII. Incertae Sedis	Eubacterium (unclassified)	3	50 (*6*.*0+/-1*.*7*)	22 (*2*.*0+/-1*.*7*)	11 (*0*.*3+/-0*.*6*)	22 (*0*.*7+/-0*.*6*)	17 (*2*.*0+/-1*.*7*)	52 (*4*.*7+/-2*.*3*)	67 (*2*.*0+/-1*.*7*)	22 (*0*.*7+/-0*.*6*)
	Lachnospiraceae	Blautia producta	4	44 (*5*.*3+/-0*.*5*)	0	0	0	0	0	0	0
		Blautia (unclassified)	194	54 (*6*.*4+/-2*.*7*)	2 (*0*.*2+/-0*.*4*)	0	0	27 (*3*.*2+/-2*.*9*)	15 (*1*.*4+/-1*.*1*)	0	0
		Ruminococcus obeum	18	35 (*4*.*2+/-2*.*1*)	4 (*0*.*4+/-0*.*5*)	0	0	23 (*2*.*7+/-1*.*9*)	19 (*1*.*7+/-0*.*7*)	0	0
		Butyrivibrio (unclassified)	17	53 (*6*.*4+/-2*.*3*)	0	0	0	21 (*2*.*5+/-3*.*9*)	14 (*1*.*3+/-1*.*3*)	0	0
		Clostridium (unclassified)	89	54 (*6*.*5+/-2*.*3*)	2 (*0*.*2+/-0*.*5*)	0	1 (*0*.*0+/-0*.*1*)	21 (*2*.*6+/-3*.*0*)	13 (*1*.*2+/-1*.*2*)	1 (*0*.*0+/-0*.*2*)	0
		Clostridium sp. SS2/1	11	31 (*3*.*7+/-0*.*5*)	0	0	0	15 (*1*.*8+/-0*.*4*)	10 (*0*.*9+/-0*.*3*)	0	0
		Coprococcus eutactus	1	33 (*4*)	11 (*1*)	0	0	50 (*6*)	44 (*4*)	0	0
		Coprococcus (unclassified)	238	50 (*5*.*9+/-3*.*0*)	1 (*0*.*1+/-0*.*4*)	0	1 (*0*.*0+/-0*.*2*)	29 (*3*.*5+/-2*.*9*)	21 (*1*.*9+/-1*.*1*)	2 (*0*.*1+/-0*.*3*)	1 (*0*.*0+/-0*.*2*)
		Dorea (unclassified)	14	52 (*6*.*2+/-1*.*0*)	0	0	0	0	8 (*0*.*7+/-0*.*5*)	0	0
		Eubacterium (unclassified)	86	53 (*6*.*3+/-2*.*2*)	2 (*0*.*2+/-0*.*4*)	0	0	20 (*2*.*5+/-2*.*3*)	12 (*1*.*1+/-0*.*8*)	0	0
		Roseburia (Eubacterium) rectale	135	54 (*6*.*4+/-2*.*4*)	5 (*0*.*4+/-0*.*5*)	0	0	24 (*2*.*9+/-2*.*5*)	17 (*1*.*5+/-1*.*1*)	1 (*0*.*0+/-0*.*2*)	0.3 (*0*.*0+/-0*.*1*)
		Roseburia (unclassified)	22	51 (*6*.*1+/-2*.*6*)	3 (*0*.*3+/-0*.*5*)	0	0	8 (*1*.*0+/-1*.*4*)	16 (*1*.*4+/-1*.*1*)	0	0
		Ruminococcus (unclassified)	41	54 (*6*.*4+/-2*.*2*)	2 (*0*.*2+/-0*.*4*)	0	0	19 (*2*.*3+/-2*.*3*)	17 (*1*.*5+/-1*.*1*)	0	0
		Eubacterium ventriosum	1	33 (*4*)	0	0	0	8 (*1*)	11 (*1*)	0	0
		Lachnospiraceae (unclassified)	1	33 (*4*)	22 (*2*)	0	0	33 (*4*)	22 (*2*)	0	0
	Ruminococcaceae	Clostridium (unclassified)	10	49 (*5*.*9+/-2*.*9*)	6 (*0*.*5+/-1*.*0*)	0	20 (*0*.*6+/-1*.*3*)	32 (*3*.*8+/-2*.*7*)	20 (*1*.*8+/-2*.*1*)	13 (*0*.*4+/-0*.*8*)	7 (*0*.*2+/-0*.*4*)
		Faecalibacterium prausnitzii	325	52 (*6*.*3+/-2*.*9*)	10 (*0*.*9+/-0*.*8*)	0	0	52 (*6*.*2+/-3*.*4*)	18 (*1*.*6+/-1*.*0*)	0	0
		Ruminococcus bromii	7	51 (*6*.*1+/-1*.*8*)	0	0	0	18 (*2*.*1+/-2*.*1*)	24 (*2*.*1+/-1*.*6*)	0	0
Gammaproteobacteria	Enterobacteriaceae	Escherichia (unclassified)	96	39 (*4*.*7+/-2*.*1*)	19 (*1*.*7+/-1*.*0*)	0	0	28 (*3*.*4+/-1*.*0*)	20 (*1*.*8+/-1*.*5*)	0	0
Erysipelotrichi	Erysipelotrichaceae	Eubacterium biforme	15	38 (*4*.*5+/-2*.*1*)	7 (*0*.*6+/-0*.*9*)	0	0	38 (*4*.*5+/-3*.*2*)	21 (*1*.*9+/-1*.*1*)	0	0
		Catenibacterium (unclassified)	12	58 (*6*.*9+/-3*.*2*)	11 (*1*.*0+/-0*.*7*)	0	0	35 (*4*.*3+/-2*.*3*)	29 (*2*.*6+/-1*.*3*)	0	0
		Clostridium (unclassified)	18	47 (*5*.*6+/-2*.*2*)	2 (*0*.*2+/-0*.*4*)	0	0	18 (*2*.*2+/-2*.*2*)	16 (*1*.*4+/-1*.*0*)	0	0
Verrucomicrobiae	Verrucomicrobiaceae	Akkermansia muciniphila	6	28 (*3*.*3+/-0*.*5*)	0	0	0	22 (*2*.*7+/-0*.*8*)	2 (*0*.*2+/-0*.*4*)	0	0

Thresholds used to establish presence from OTU binary data are presented in the methods section. The number of samples collected for each stage at each location is noted in parentheses after the stage name. The number of OTU per taxon used to generate the table is noted after each taxon name. When there was only one OTU in a taxon in this dataset, the number of samples containing that OTU is shown in parentheses. When a taxon was represented by more than one OTU, the mean and standard deviation were calculated for the number of positive samples per stage. The number or mean number of positive samples was converted to a percentage to allow comparison across stages with different numbers of samples. Values shown are percentage (mean count +/-SD).

**Fig 2:**
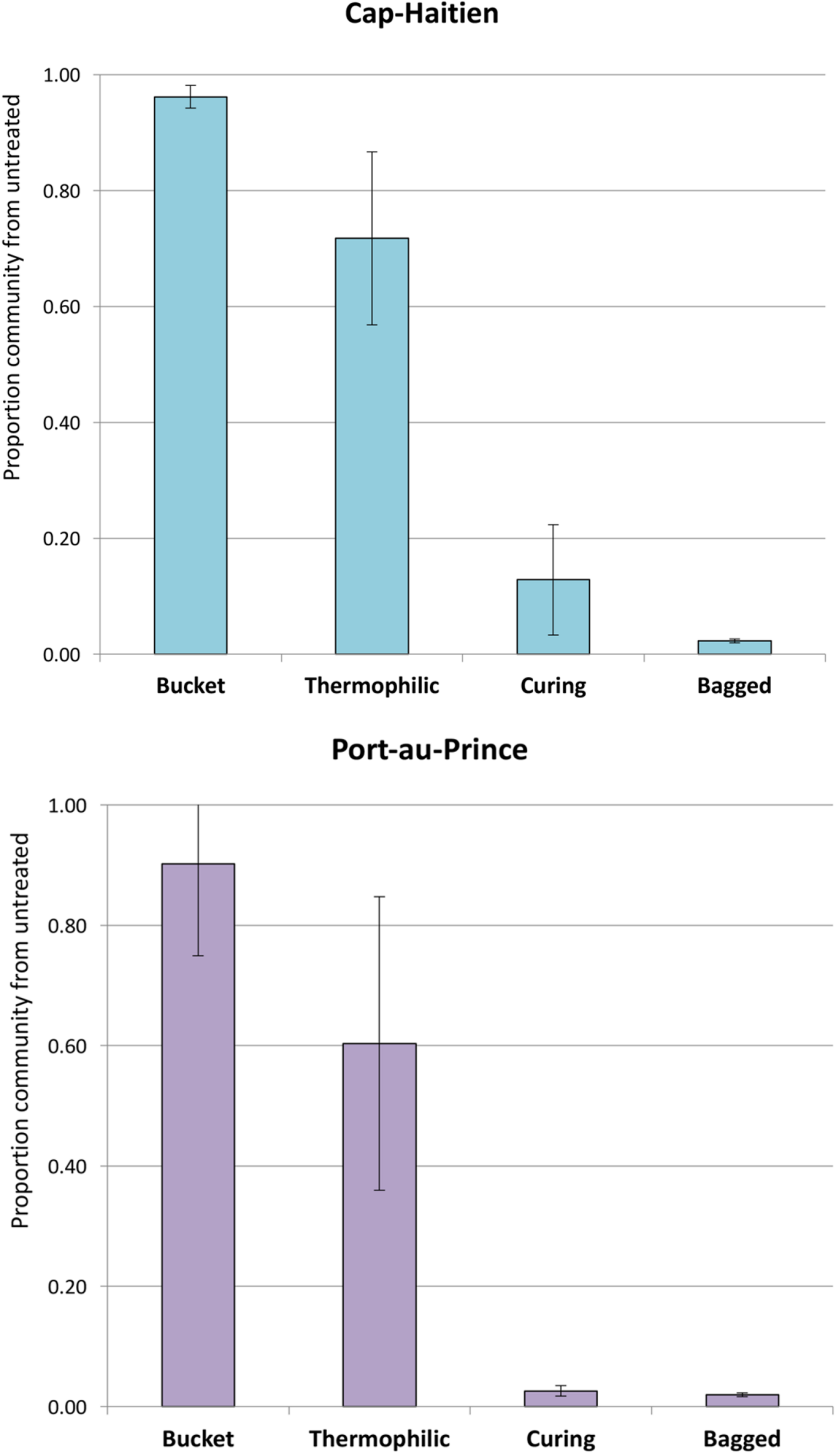
Proportion of microbial community derived from untreated source material (Bucket) samples throughout the composting process. Proportions were estimated by SourceTracker using binary OTU data from individual Bucket samples as the training (source) set and Thermophilic, Curing and Bagged samples as sinks. Source proportions in each Bucket sample were estimated using the leave-one-out validation procedure and averaged. Error bars are standard deviations around the mean.

To track the fate of the broadest set of organisms from the Bucket samples, the SourceTracker analysis used binary data from all OTU called present in the Bucket samples. SourceTracker analysis indicated that the majority of OTU richness detected at the thermophilic stage was derived from the Bucket samples (Fig 2). By the Curing stage, only 13% and 3% of OTU richness in Cap-H and PauP, respectively, was derived from the initial Bucket community, and further reduced to 2% at the Bagged stage for both sites. This indicates that the microbial community found at the Bucket stage was almost completely replaced by other bacteria by the Bagged stage.

### Potentially pathogenic bacteria

In addition to assessing the fate of general human-fecal-associated bacteria, we examined the data for OTU classified specifically within potentially (opportunistically) pathogenic bacterial species–such as *Shigella dysenteriae*, *Vibrio cholera*, and *Escherichia coli*–which are of particular concern for internally displaced persons camps in Haiti. There were very few OTU in the main dataset from any stage that were classified specifically as one of the species of greatest health concern. To determine if we missed samples with OTU called present in fewer than five samples across the entire dataset (the initial cutoff for an OTU to be included in the main dataset of 54 samples, which yielded 7531 OTU), the complete dataset of 19,994 OTU was examined for OTU classified in the species of interest. No OTU were classified specifically within the following species (according to the 2011 PhyloChip taxonomy, which is based on an alignment of high-quality 16S rRNA gene sequence of at least 1300 nucleotides in length): *Mycobacterium tuberculosis*, *Clostridium difficile*, *Escherichia coli*, *Shigella dysenteriae*, or *Vibrio cholera*. There was one *Mycobacterium leprae* OTU detected in one Curing sample (PauP), one *Clostridium tetani* OTU detected in four Bucket samples (1 Cap-H, 3 PauP), one Thermo sample (PauP), and two Bagged samples (PauP), and several *Serratia marcescens* OTU called present in at least one sample at the Bucket, Thermo, or Curing stage (none at the Bagged stage) (Table 2, top panel). Some of the sequences included in the current PhyloChip taxonomy as unclassified at the species level had originally been deposited in gene sequence databases with designations such as *Escherichia coli* (11 OTU), *Salmonella* subsp. *enterica* serovar Typhimurium (9 OTU), and *Shigella dysenteriae* (1 OTU). When aligned to sequences verified at the species level for the PhyloChip taxonomy (based on the Greengenes taxonomy), the sequences fall outside the species-level classification, but can serve here as proxies for these target organisms.

**Table 2:** Average percentage of samples in each composting stage with an opportunistic pathogen species or near-neighbor OTU called present using thresholds defined in the methods section.

	# OTU averaged	Cap-Haitien				Port au Prince			
Species of interest		Bucket (12)	Thermo (9)	Cured (3)	Bagged (3)	Bucket (12)	Thermo (9)	Cured (3)	Bagged (3)
Mycobacterium leprae	1	0	0	0	0	0	0	33 (*1*)	0
Clostridium tetani	1	8 (*1*)	0	0	0	25 (*3*)	11 (*1*)	0	67 (*2*)
Escherichia unclassified (E. coli)	11	23 (*2*.*8+/-2*.*3*)	17 (*1*.*5+/-1*.*4*)	0	0	23 (*2*.*7+/-1*.*8*)	16 (*1*.*5+/-1*.*9*)	0	0
Salmonella enterica (serovar Typhimurium)	9	24 (*2*.*9+/-1*.*8*)	0	0	0	30 (*3*.*6+/-1*.*7*)	0	0	0
Serratia marcescens	11	16 (*1*.*9+/-2*.*3*)	1 (*0*.*1+/-0*.*3*)	24 (*0*.*7+/-0*.*6*)	0	19 (*2*.*3+/-2*.*5*)	2 (*0*.*2+/-0*.*4*)	0	0
Shigella unclassified (Shigella dysenteriae)	1	0	0	0	0	17 (*2*)	0	0	0
**Genus (proxy for species of interest)**									
Mycobacterium	23	1 (*0*.*1+/-0*.*3*)	2 (*0*.*2+/-0*.*6*)	20 (*0*.*6+/-1*.*0*)	55 (*1*.*7+/-1*.*3*)	9 (*1*.*1+/-1*.*4*)	5 (*0*.*4+/-1*.*1*)	88 (*2*.*7+/-0*.*5*)	80 (*2*.*4+/-0*.*9*)
Clostridium (Order Clostridiales, Family Clostridiaceae)	132	40 (*4*.*8+/-3*.*1*)	7 (*0*.*6+/-0*.*8*)	0.3 (*0*.*0+/-0*.*1*)	3.5 (*0*.*1+/-0*.*4*)	41 (*4*.*9+/-3*.*4*)	27 (*2*.*5+/-1*.*9*)	22 (*0*.*6+/-0*.*9*)	24 (*0*.*7+/-1*.*1*)
Escherichia	96	39 (*4*.*7+/-2*.*1*)	19 (*1*.*7+/-1*.*0*)	0	0	28 (*3*.*4+/-1*.*0*)	20 (*1*.*8+/-1*.*5*)	0	0
Salmonella	13	31 (*3*.*8+/-2*.*2*)	0	0	0	31 (*3*.*7+/-1*.*1*)	0.9 (*0*.*1+/-0*.*3*)	0	0
Serratia	6	36 (*4*.*3+/-2*.*3*)	1.9 (*0*.*2+/-0*.*4*)	28 (*0*.*8+/-0*.*8*)	0	35 (*4*.*2+/-1*.*7*)	3.7 (*0*.*3+/-0*.*5*)	0	0
Shigella	22	39 (*4*.*6+/-1*.*4*)	0	0	0	57 (*6*.*8+/-0*.*4*)	8 (*0*.*7+/-0*.*5*)	0	0
Vibrio	3	39 (*4*.*7+/-5*.*0*)	0	22 (*0*.*7+/-0*.*6*)	44 (*1*.*3+/-1*.*5*)	44 (*5*.*3+/-3*.*1*)	15 (*1*.*3+/-1*.*5*)	56 (*1*.*7+/-1*.*2*)	0

The number of samples collected for each stage at each location is noted in parentheses after the stage name. The number of OTU for each species of interest is noted after the organism name. When there was only one OTU in a taxon in this dataset, the number of samples containing that OTU is shown in parentheses. When a taxon was represented by more than one OTU, the mean and standard deviation were calculated for the number of positive samples per stage. The number or mean number of positive samples was converted to a percentage to allow comparison across stages with different numbers of samples. Values shown are percentage (mean count +/-SD).

Broadening the search to include additional near-neighbor OTU–all those within the same genus from the 7531 OTU dataset- allowed further evaluation of proxies for opportunistic pathogens, though mostly they were OTU designated ‘unclassified’ at the species level. Results are presented in the genus-level data in Table 2, showing a decline in relative richness at the Thermo stage for all but *Mycobacterium* at Cap-H. The enteric bacteria (within genera *Escherichia*, *Salmonella*, *Serratia*, *and Shigella*) were detected in roughly a third to one half of the samples at the beginning (Bucket), and declined to undetectable levels by the end of the process (Bagged) at both locations. In contrast, OTU within *Mycobacterium* and *Vibrio* increased after the Thermo stage and remained in a greater percentage of samples, except for *Vibrio* at PauP.

### Microbial community dynamics

To evaluate bacterial community changes throughout the composting process, we identified 7531 OTU present in at least five samples from the combined categories of Bucket, Thermophilic, Curing, and Bagged compost (from either location, 54 total samples). The binary data (presence/absence calls) were used to assess relative richness at each stage. Various bacteria comprised these phyla in the different stages, yet it is clear the heat and other environmental conditions of the thermophilic stage reduced community complexity of the starting material at both locations (Fig 1B). OTU relative richness decreased the most from Bucket to Thermo stages at both locations, as stated earlier (p<0.0001 for Cap-H, p<0.001 for PauP) (Fig 1A). The average relative richness remained low at the Curing stage at Cap-H, yet the final relative richness (Bagged samples) was similar between the two locations. Of the 7531 OTU in the entire set, 297 were called present only in the Curing or Bagged samples–and 39.7% of those were affiliated with *Streptomyces*. There were a few genera (e.g., *Rhodanobacter*, *Microbulbifer*, *Nitrosococcus*, *Actinomyces*, *Sorangium*, *Dietzia*) and a couple species (*Nitratireductor aquibiodomus* and *Actinomadura vinaceae*) that were called present only in the Curing and Bagged samples, but mostly the bacteria in Curing/Bagged samples had been called present in earlier stage samples, too.

**Fig 3:**
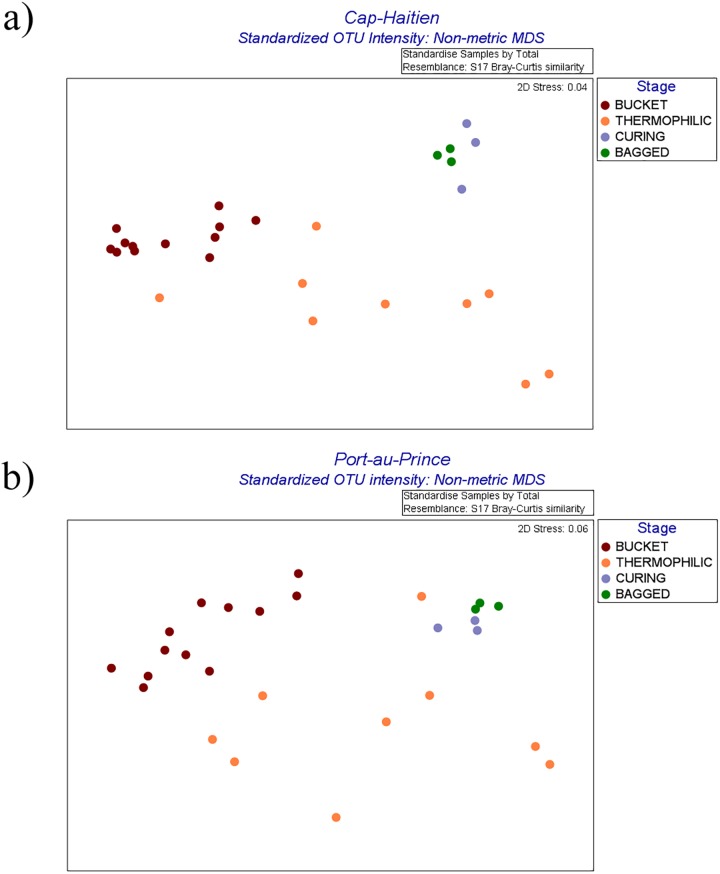
Non-metric multidimensional scaling (NMDS) plots of Bray-Curtis similarity matrices based on standardized OTU intensities. Number of samples at each stage at each location were: Bucket (12), Thermophilic compost (9), Curing (3), Bagged (3). a) Cap-Haitien, b) Port-au-Prince

**Fig 4:**
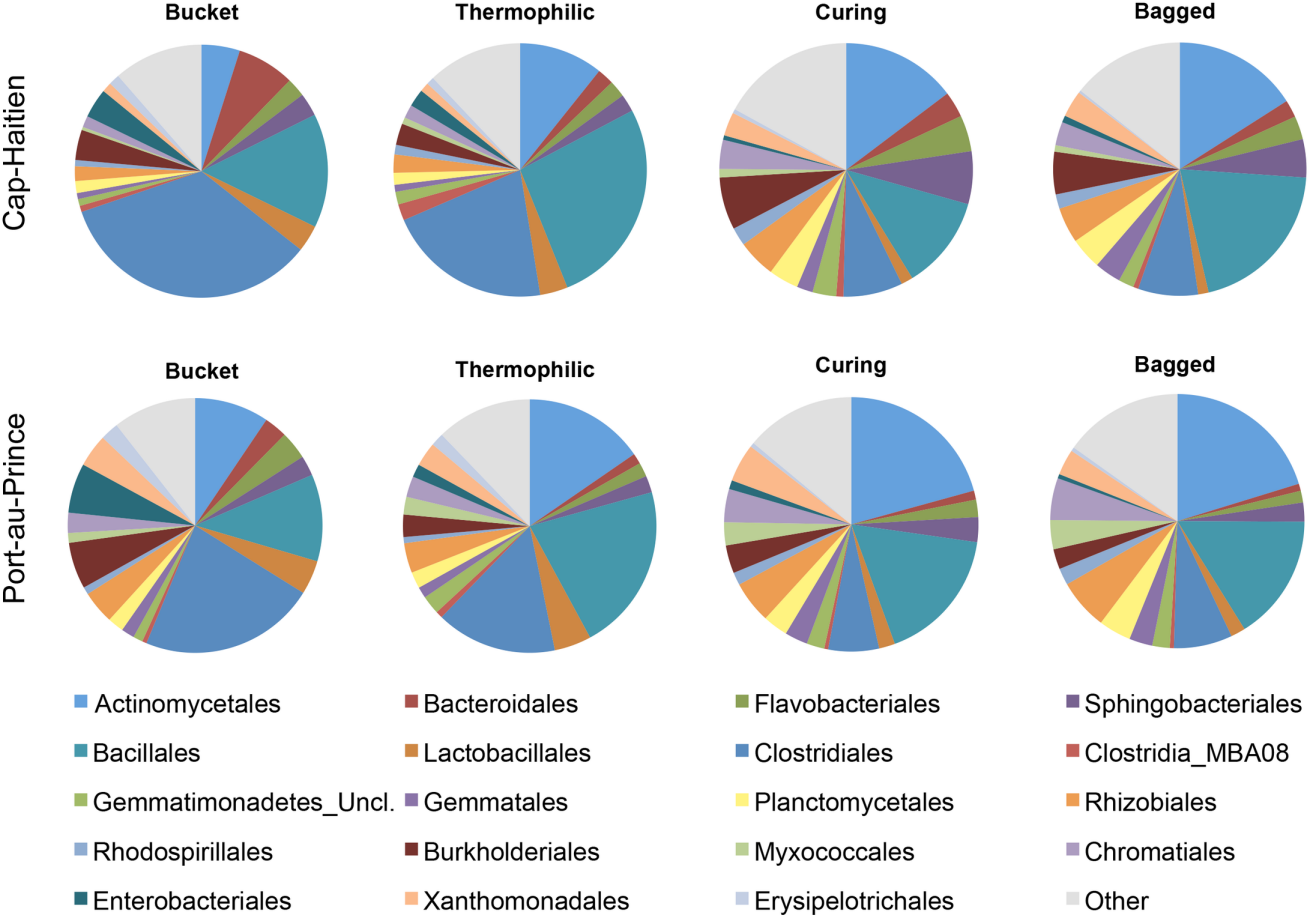
Pie charts show a summary of bacterial orders containing OTU that changed the most in relative abundance (probe intensity scores) through the thermophilic composting process. OTU for each location were included if they were within the top 100 OTU changing the most (for all pairwise comparisons within a location) and/or had at least a 10-fold change in average abundance across compost stages within each location. OTU within the orders could differ by location. Average intensity values were calculated for each OTU and percent contributions were summed at the order level for each stage and location. Named orders are those contributing at least 2% at any compost stage in either location. Following pie charts clockwise, names are listed in the legend from left to right.

While microarray analysis provides some insight about relative richness, its strength lies in assessing changes in relative abundances of community members across a sample set. The PhyloChip analysis can track changes in bacterial populations over four orders of magnitude by comparing OTU probe set intensity values [27]. For the current study, the OTU probe intensity data were used to identify bacterial groups changing the most in relative abundance from the source material through to the finished product (bagged compost). Non-metric multidimensional scaling (NMDS) plots based on OTU intensity values reflect the structural changes during composting and highlight the variability among samples at the beginning of the process and the relative similarity in bacterial community structure in the finished compost (Fig 3). Analysis of Similarities (ANOSIM) showed very clear differences between the starting material composition and the finished product at each location (Global R was 0.626 for Cap-H and 0.623 for PauP). Pairwise ANOSIM R statistics were at or near one when Bucket samples were compared to Curing or Bagged samples, which signify very different community structures after composting (S5 Table). Detailed information regarding which bacterial taxa changed the most in relative abundance throughout the composting process is presented in S6 Table and S7 Table (SIMPER, top 5% contributing OTU are presented) and partially summarized in S1 Table and S2 Table as data bars. The information is further summarized at the order level by percent contribution to summed intensities in Fig 4 (and S10 Table contains Kruskal-Wallis and posthoc p-values for those orders). SIMPER between-group dissimilarity values are appended to S5 Table for ease of cross-reference. For both locations, the greatest variability in community structure was among Thermo samples (84% similarity at Cap-H, 86% similarity at PauP, followed by the Bucket samples (92% sim. at Cap-H, 91% at PauP), and Bagged samples had the greatest similarity (96% at Cap-H, 97% at PauP).

At both locations (but especially at PauP) Enterobacteriales OTU decreased significantly between Bucket and Bagged stages (S10 Table). Members of the Bacillales (especially *Geobacillus*) increased the most at the thermophilic compost stage, but then decreased at the Curing stage before rebounding in the final stage. The SIMPER analysis also highlighted the increased relative abundance of the Bacillales at the Thermo stage at PauP, along with a few Actinomycetales, Clostridiales, and Gemmatimonadetes. The Bacteroidales decreased notably in the Thermo stage (Fig 4, S10 Table), whereas the Lactobacillales differed significantly only at Cap-H (S10 Table). The Burkholderiales differed significantly at both locations from their initial values (S10 Table). From the additional SIMPER information, it was observed OTU in Lactobacillales (*Enterococcus* and *Streptococcus*) and Burkholderiales (*Aquabacterium* and *Diaphrobacter*) increased in Thermo samples at Cap-H. The Planctomycetales and all orders in the Proteobacteria, save the Enterobacteriales (both locations) and the Erysipelotrichales (PauP), increased in percent contribution in the Curing/Bagged stages. Unclassified OTU affiliated with Cellulomonadaceae increased in both locations at the Curing stage, though they peaked in the Bagged stage at Cap-H. The same trend was observed for Thermomonosporaceae (S1 Table and S2 Table).

### Genus-level observations

#### Cap-Haitien

Despite the observed decline for almost all OTU within the Clostridiales at the Thermophilic stage, an OTU affiliated with *Symbiobacterium thermophilum* (in the Clostridiales) showed the greatest fold-increase (5.8-fold (215 to 1252 FI units)) between the Bucket and Thermo samples at Cap-Haitien, whereas members of the Bacillaceae (notably several *Geobacillus*) had the greatest increases in mean FI units (4045 to 6329 FI unit increases). As noted in [32], a doubling of intensity is roughly equal to more than a four-fold increase in relative abundance. OTU within *Planifilum* (Family Thermoactinomycetaceae within the Bacillales) also had large relative abundance increases at the Thermo stage (4146 FI units). The OTU with the very greatest intensity increase (# 47170) consisted of two sequences: one previously amplified from compost (GenBank accession #AB034710) and one sequence from a sewage sludge isolate, noted as being a thermophile and named *Bacillus thermocloaceae* (55°C optimal growth; [58,59]). Several members of the Actinomycetales (e.g., *Thermobifida*, *Saccharomonospora*, *Streptomonospora*, *Psuedonocardia*, and *Actinomadura*) also increased in the Thermo stage (S1 Table; corroborated by SIMPER results (S6 Table)), and whereas the members of the Bacillales decreased in the Curing stage, those members of the Actinomycetales continued to increase. A few unclassified OTU within the Rhodospirillaceae also increased in both the Thermo and Curing stages. Notably, OTU in two genera of Actinomycetales (*Arthrobacter* and *Streptomyces*) declined at the Thermo stage, but rebounded in the Curing and Bagged stages. Members of the Flavobacteriaceae (e.g., *Chryseobacterium* and *Flavobacterium*), Chitinophagaceae (*Flavisolibacter*), and Saprospiraceae followed the same trend as the latter actinomycetes, as did the Sphingobacteriaceae (*Parapedobacter* and *Sphingobacterium*). Most of the Gammaproteobacteria declined in the Thermo stage and rebounded in the Curing and Bagged stages, with the following exceptions: *Succinivibrio*, *Escherichia*, *Klebsiella*, *Shigella*, and unclassified Enterobacteriaceae, all of which declined in the Thermo stage and even more so in the Curing stage with only small increases thereafter. These observations were largely corroborated in SIMPER results using only the top five percent of OTU contributing to dissimilarities between stages (S6 Table).

An unclassified member of the Conexibacteraceae increased in the Bagged samples, from roughly 2000 to nearly 9000 FI units (S1 Table). The type strain of *Conexibacter* (*C*. *woesei*) was isolated from soil [60], is an aerobe that is also capable of reducing nitrate, and is saccharolytic, likely contributing to both carbon and nitrogen cycling. Several taxa commonly associated with soil also increased in the Curing stage and had equal or greater relative abundance in the Bagged stage: unclassified members of Class Gemmatimonadetes, Families Gemmataceae, Isosphaeraceae, Planctomycetaceae (e.g., *Planctomyces*), and various members of the Order Rhizobiales. The same trends are true for most of the OTU in the Burkholderiales, though SIMPER results show greater abundance of *Bordetella* OTU in Curing samples relative to Thermo samples. This is consistent with *Bordetella* being mesophilic. Notably, a *Methyloversatilis* and an unclassified Methylophilaceae increased nearly 2- to 10-fold the Curing phase and then dropped or remained even in the Bagged stage samples. This is in contrast with *Methylocaldum*, which after doubling at the Curing phase, then peaked in the Bagged samples.

#### Port-au-Prince

The bacteria that changed the most throughout thermophilic composting at PauP are largely the same as observed at Cap-H. Many members of the Clostridiales declined at the Thermophilic stage–just less dramatically and consistently—but *Symbiobacterium thermophilum* increased 36-fold. Several other genera also had double-digit fold-increases: *Geobacillus* (29-fold), *Bacillus* (24-fold), an unclassified Thermomicrobium/Sphaerobacter (21-fold), and a *Paenibacillus* (14-fold). The increase of *Thermobifida fusca* at the Thermo stage was even greater at PauP than at Cap-H (10720 FI units) and was the species with the greatest relative abundance increase at PauP. An unclassified *Desulfotomaculum* also increased markedly at this stage, as did unclassified Thermodesulfobacteriaceae. Other genera with notable relative abundance increases at the Thermo stage belonged to the Bacillaceae (*Paenibacillus*, *Bacillus*, *Geobacillus*, *Natronobacillus*), Planococcaceae (*Ureibacillus*), and Thermoactinomycetaceae (*Planifilum*). These OTU increased anywhere from 4600 to 8698 FI units, almost always showing a greater increase in PauP samples than was observed for the Cap-H samples. Generally, members of the Bacillales maintained their relative abundance values in the Curing stage at PauP, whereas they had declined somewhat at Cap-H. These observations are corroborated by the SIMPER analysis (S7 Table).

Conversely to the decline of *Arthrobacter* and *Streptomyces* observed at Cap-Haitien, members of these genera remained stable during the Thermo stage before increasing at the Curing stage at PauP. Unclassified OTU in the genus *Corynebacterium* had similar intensity values in Bucket and Thermo stages at both locations, but whereas they then decreased at Cap-Haitien, they either remained similar or increased in later stages at PauP. There was a greater increase in relative abundance for OTU affiliated with *Actinomadura* at PauP than was observed at Cap-H. The SIMPER analysis highlighted a difference for a few OTU within *Faecalibacterium* (including *F*. *prausnitzii*) whereby there was greater relative abundance in the Bagged than Curing samples at PauP, whereas *Faecalibacterium* OTU were not even included in the list of top five percent of OTU contributing to differences between these two stages at Cap-H (they were consistently low in both). Within the Xanthomonadaceae, OTU classified to genus *Ignatzschineria* (associated with fly larvae) decreased substantially at the Thermo stage and then remained about steady for the remainder of the process. In general, members of the Xanthomonadaceae (e.g., *Dyella*) fared better during the Thermo and Curing stages at PauP than at Cap-H. The increases in methylotrophic bacteria between Thermo and Curing were more muted than at Cap-H, except for *Methylocaldum*, which still doubled at the Curing phase before declining in Bagged samples.

## Discussion

### Fate of human-associated bacteria during thermophilic composting

Molecular methods for DNA-based assessment of microbial community composition allows for a comprehensive tracking of all fecal organisms throughout the composting process. To assess the fate of fecal bacteria at SOIL composting facilities, we employed a high-density DNA microarray. A notable benefit of this approach was being able to better define the starting fecal material and tracking the fate of many bacteria simultaneously. We noted that while the Bucket samples contained typical human fecal bacteria, there were fewer OTU classified in the various *Bacteroides* species than reported for fecal material from certain populations (S3 Table). Using the 75 non-redundant species with at least 1% genome coverage (by Illumina sequencing) reported by Qin and colleagues [9], the ‘common core’ for European individuals consists of roughly 32% Bacteroidaceae, the family with the greatest percentage of species. Having fewer Bacteroidaceae, specifically *Bacteroides*, representatives in the Bucket samples than observed by Qin and colleagues may be due to one or more of several reasons. One possible reason is a difference in diet [61–66]. Although we did not specifically test freshly voided feces (and we are not aware of any published reports on health, adult, Haitian gut microbiota profiles), Ruminococcaceae (especially *Faecalibacterium*) and Lachnospiraceae are typically reported as part of the gut microbiota and serve as useful markers for this study. It is possible the Haitian diet or yet-unknown regional influences support more Ruminococcaceae and Lachnospiraceae relative to Prevotellaceae and especially Bacteroidaceae (1482, 1367, 309, and 18 OTU, respectively, in the full dataset; 617, 362, 10, and 2 in the subset of 1768 human-associated OTU) as has been reported for other populations [61,63,67]. Additionally, there may have been changes that occurred between feces deposition and bucket collection (up to two weeks). The extended lag time between deposition and bucket collection combined with bagasse addition after each deposition may have resulted in greater oxygen penetration through the material collected in the buckets than otherwise would have been the case, potentially reducing the relative proportions of these strictly anaerobic, non-spore-forming bacteria. The use of urine-diverting toilets also would be expected to reduce the anaerobicity of the deposited material, as liquid (which impedes oxygen exchange) was shunted away. Finally, the buckets in the field typically are exposed to fairly high ambient temperatures, which may have altered the original composition or structure. Despite the reduced number of representative Bacteroidaceae–which may be indicative for this population, the Bucket samples clearly showed a definitive fecal signal, which included members from both ‘Westernized and non-Westernized’ gut profiles previously reported (e.g. *Bifidobacterium*, *Coprococcus*, *and Blautia* being more abundant in Westernized population profiles and *Catenibacterium*, *Spirochaetaceae*, *Prevotella* being more prominent in Non (or less)-Westernized population profiles [62,64–68]). Ruminococcaceae (esp. *Faecalibacterium*) and Lachnospiraceae are generally well-represented in any fecal sample, so we believe the taxa used for comparison in Table 1 are adequate to demonstrate one of the three ways used to show reduction in relative abundance of fecal microbiota through thermophilic composting.

When evaluating the fecal-associated OTU as defined by the Bucket samples (Fig 1C), by Qin et al.’s common core (Table 1), or by all Bucket sample OTU (SourceTracker), the data show a substantial decline throughout the composting process at PauP and Cap-H, with only a slight rebound at Cap-H. Finstein and colleagues [69] cautioned having too great a decline in the microbial community at the start of the composting process leaves an abundance of readily accessible nutrients available for rapid regrowth when conditions become more favorable, which may have been what caused the apparent rebound observed in Cap-H samples. Cap-H piles also may retain more of the original nutrients because leachate is returned to the piles rather than passing into the soil, as would occur at PauP. Additionally, storage facilities for the bagged compost also differ in that Cap-H storage is shaded. Alternatively, it is possible the apparent rebound observed was due to different piles being sampled for the various stages, rather than actual regrowth being observed in piles over time. The few species of the common core called present in the Bagged samples were predominantly spore-formers (e.g., *Clostridium* sp.). Their presence at this stage could be due to spores being present (and lysed during DNA extraction), regrowth from spores, or from a re-introduction to the piles after the thermophilic stage, as the piles are open-air throughout the composting process.

The fate of the Enterococci, often used as fecal contamination indicators, differed between locations, especially at the thermophilic stage. We noted they declined more rapidly at Cap-H than at PauP. The steeper decline may have resulted from what arguably was a more stringent environment due to suspected elevated ammonia levels at Cap-H (see discussion below regarding pile construction and ammonia levels). While the enterococci are hardy, withstanding desiccation, pH extremes, oxidative stress, antibiotics, and survive over a wide range of temperatures (10–45 ^o^ C) [14], the effect of excess ammonia has not been reported. The apparently less stressful (i.e., lower ammonia) environment of the PauP piles may be the reason for increased detection of *E*. *faecalis* and *E*. *casseliflavus* there. In fact, two OTU within *E*. *casseliflavus* and three OTU within *E*. *faecalis* had slightly greater intensities in Thermo samples at PauP, whereas all those OTU decreased at Cap-H at the same stage. Conditions at Cap-H also could have been favorable for modest regrowth, resulting in slightly greater relative abundances in Bagged versus Cured compost (S4 Table). Tønner-Klank and colleagues [8] observed regrowth of enterococci in later samples of composted waste and suggested the practice of using culturable enterococci to evaluate the fate of fecal organisms more generally (i.e., as indicator organisms) may over-estimate total fecal organism populations.

While the current study is the only study to date we are aware of to track the bacterial community from raw fecal material throughout a thermophilic composting process, other studies that tracked bacterial changes during sewage sludge composting show some similarities. Ishii and Takii using DGGE analysis [3] observed rapid changes from sewage sludge starting material after it was added to an Earp-Thomas composting system and then saw fairly stable patterns for the next 10 days. They noted *Bacillus*, *Clostridium*, an actinobacterium, and Gram-negative bacteria (e.g., *Thermomicrobium roseum*) were prominent members of the early composting process. Though one member (*B*. *thermocloacae*, affiliated with two sequences) also has been isolated from sewage sludge, most of the recovered sequences were affiliated with bacteria commonly found in a variety of composting samples. The limited resolution of the analytical method (DGGE) and short study period (10 days) prevented more detailed tracking of the fate of the sewage sludge bacteria. Su et al. [25] used a high-throughput iTag sequencing method to follow sewage sludge through a bench-scale thermophilic (>55°C for two days) composting process, which revealed reductions of Proteobacteria, Nitrospirae, and Actinobacteria in the first two days, as the system established thermophilic conditions. The Chloroflexi and Bacteroidetes, also prevalent in sewage sludge, were substantially reduced by Day 8, when the compost was starting to cool. Members of the Proteobacteria declined during the thermophilic phase in both Su and colleagues’ experiment and the current study, yet we observed variable trends at the phylum level for Firmicutes. They observed substantial increases, while we observed substantial decreases, but in both cases, representatives of the Bacillales increased at the thermophilic stage. It may be the other bacteria that constituted a large proportion of the Firmicutes in the raw fecal samples analyzed in the current study had already been lost from the dewatered sewage sludge used by Su et al. as starting material.

### Potentially pathogenic bacteria

From the perspective of human health and the environment, overall bacterial community reduction is not an objective (in fact a healthy bacterial community is an indicator of good compost); it is pathogen reduction that is critical. Previous studies have shown that failing to obtain even moderately thermophilic conditions leads to prolonged survival of fecal indicators, observed in several studies of composting toilets [6–8]. Prolonged survival may occur because the original host body temperature is warmer than typical environmental temperatures and so the compost must reach thermophilic conditions throughout the pile for a sufficient length of time to reduce the potential pathogen load. Furthermore, the work of Marin et al. [70] underscores the difficulty in eradicating some pathogens, in their case shigatoxigenic *E*. *coli*, when composting occurs for less than two months, even when reaching 61°C (non-aerated piles) or 66°C (aerated piles) during some portion of that time. Pourcher et al. [4] observed a 4 log_10_ decrease of *E*. *coli* and also of enterococci in composted sewage sludge after four months of composting, although there was an initial slight increase in culturable counts, despite the hottest part of the pile reaching 66–69°C. Feachem *et al*. [71] provides pathogen time-temperature ‘death curves’ for *Shigella*, *Salmonella*, *Vibrio cholerae*, in addition to *Ascaris*, *Taenia*, and *Entamoeba histolytica* and note that enteroviruses and *Ascaris* eggs are the most hardy.

When Berendes et al. [37] evaluated *Ascaris* ova and *E*. *coli* viability in samples collected from the PauP SOIL facility (sampled during 2012 and from piles that were not turned periodically), it took 75 days (nearly 11 weeks) to observe a 4 to 5 log_10_ reduction in viable *E*. *coli*. This reduction resulted in no detectable viable *E*. *coli* using a modified Colilert protocol (LOD was 142 MPN/g dry wt.) in the remaining samples evaluated (age of sample up to 330 days). While the current study was not undertaken specifically to compare culturable fecal indicator counts to microbial community changes detectable by the PhyloChip DNA microarray, we provide MPN measurements from two bins still receiving input material that had high starting concentrations of *E*. *coli* during Fall 2013 at Cap-H. The same bins/piles were retested throughout compositing, and counts declined from roughly 7.2x10^4 and 2.4x10^6 to less than 1x10^3 MPN in both cases within 13 and 42 days, respectively (S8 Table). Importantly, an additional 38 tested bins/piles had fewer than 5.2 x10^3 MPN when first tested, 30 of which had <1x10^3 MPN. These ancillary data support the conclusion of Enterobacteriaceae population reductions during thermophilic composting indicated by the microarray results. Furthermore, Dubinsky and colleagues [36] demonstrated *E*. *coli* and enterococci counts correlate with average PhyloChip OTU probe intensities of *Escherichia* OTUs (r^2^ = 0.73) and *Enterococcus* OTUs (r^2^ = 0.46), respectively, further supporting the conclusions reached about observed reductions for the compost samples.

After the Berendes et al. [37] study results were known to SOIL administrators, the composting procedure was modified to incorporate turning to assure edge material, which had lower temperatures than center material, was folded into the center region at each turning. This was expected to further reduce any possible regrowth of potentially pathogenic bacteria. We looked broadly at the data from our study for the presence of known opportunistically pathogenic bacteria and found only a few OTU classified within the species-level taxa of interest, so we then evaluated trends for OTU within the genera of interest (mostly unclassified at the species level). Based on the results presented herein, the current thermophilic composting process was effective in reducing opportunistic pathogen load when the compost was allowed to complete the curing process, the complete process taking approximately one year after pile construction. While there were increases in *Mycobacterium* and *Vibrio* OTU in the Curing and/or Bagged stages relative to Bucket samples, members of these genera are commonly found in environmental samples and may not pose a health risk. For example, members of the genus *Mycobacterium* are commonly associated with plants and soils [72,73]. The results of this study are consistent with those reported by Pourcher et al. [4] for substantial reductions of enteric bacteria and with those of Ceustermans et al. [74] regarding inactivation of *Salmonella* during composting. Of the nine OTU affiliated with *Salmonella enterica* serovar Typhimurium and an additional four OTU within the genus *Salmonella*, none were called present from the thermophilic stage onward. Ceustermans and colleagues observed inactivation (no growth) after 10 hours at 60^o^ C, and while *Salmonella* survived longer at lower temperatures, it was not viable after one week at >50°C or two weeks at >40°C. Of the studies reviewed by Ceustermans et al., *Salmonella* did not survive longer than 60 days in any system that obtained at least moderately thermophilic conditions (>40°C for at least part of the process).

Only one potentially pathogenic OTU (affiliated with *Clostridium tetani* at the species-level of classification) was detected in any Bagged compost sample. The natural habitats of *C*. *tetani* are soil, dust, and intestinal tracts of animals [75], so the presence of bacteria closely affiliated with this species is not surprising, given the compost piles are open to the air and are in contact with the soil at PauP. Also, because *Clostridium* spp. are strict anaerobes, they are unlikely to pose a substantial public health risk being so the compost is naturally aerated during the application process. It is important to note we did not assay for the plasmid sequence (*tet*X) that provides for tetanus toxin production [76], so it may be the representatives of this species detected in the samples are not capable of producing the toxin, yet fall within this species designation based on the 16S rRNA gene probe sequences included on the microarray. The genus-level data in Table 2 shows several OTU affiliated with *Clostridium* other than species *tetani* are present in the later stages of compost at PauP.

The third generation PhyloChip (G3) can detect 16S rRNA gene amplicons present at roughly 1.85 x 10^7^ gene copies in a background of many other bacterial amplicons (S8 Table of [28]), which theoretically can be obtained with 10 copies of a sequence after 25 cycles of PCR, using a conservative amplification efficiency of 1.8 (a perfect doubling of gene copies every cycle would yield an amplification efficiency of 2). This detection sensitivity combined with the breadth of bacterial sequences probed simultaneously are primary reasons why PhyloChip is a useful tool for following reductions in fecal-associated bacteria through the composting process. Dubinsky *et al*. [36] have proposed that a bacterial community based approach to monitoring fecal signals in environmental samples provides a robust measure of potential contamination relative to traditionally used fecal indicator bacterial tests, somewhat akin to what was demonstrated using whole-community bacterial signatures for observing recent influences of environmental contaminants in various ecosystems [77].

### Microbial community dynamics

Thermophilic composting piles reach very hot temperatures, typically 60–70°C, within a few days of pile construction and may remain above 55^o^ C for a month or more [37,78]. The heat is the result of intense microbial metabolic activity [79], yet many bacteria in the starting material cannot withstand the high temperatures generated during the thermophilic composting stage, resulting in a decrease in overall richness, diversity [18,25], and total culturable counts [80,81]. We observed decreased relative richness in the binary data consistent with these earlier reports. Multiple studies have documented bacterial community composition changes as compost temperature rises and falls [21–23,79,80]. Competitive interactions among organisms also influence community dynamics [69]. For example, actinomycetes, notably *Streptomyces*, are known to produce a variety of antibiotics as secondary metabolites. While there have been a few detailed analyses of bacterial community structure throughout the composting process, the majority of studies to date have commonly used either culture-based analysis [79,80,82] or low-resolution molecular techniques (e.g., sequencing clones [15,16] or DGGE analysis [3,18–20]). Only two recent studies have used higher-resolution sequencing methods to evaluate sewage sludge compost [25] or zoo compost [26], and Rawat and Johri [78] have reviewed the literature available before 2013. Compost derived from raw human waste, though, has yet to be examined from starting material to finished product.

Concurrent with the decreases for most fecal bacteria discussed previously, there were notable increases in OTU classified within Firmicutes (*Bacillus*, *Geobacillus*, *Ureibacillus*, *Planifilum*, etc.) and various Actinobacteria (*Thermobifida fusca*, *Saccharomonospora*, *Streptosporangium*, etc.) at the thermophilic stage. Increases for these bacteria are common during thermophilic composting [78]. Bacteria within these taxa have an array of enzymes for plant wall decomposition and can be thermotolerant or thermophilic to various degrees [82–84]. Whereas most Lachnospiraceae OTU classified within species that are signature fecal Firmicutes (within the Clostridiales) declined precipitously at the thermophilic stage, indicating a lack of thermotolerance–and perhaps an intolerance of other environmental factors—several Lactobacillales OTU did not decline until later. Previous studies also have reported Lacobacillales members in compost detectable into the thermophilic stage [3,16]. There were also actinomycetes that had a delayed increase (until the Curing phase and even into the Bagged phase), such as *Arthrobacter*, *Streptomyces*, and *Actinomadura*, which is typical, as most members of these genera are mesophilic. Several Proteobacteria associated with plant compound degradation (e.g., lignin degradation by a xanthomonad) also increased in the later composting stages. The similarity of organisms with elevated relative abundance in the Curing and Bagged samples to compost produced from other starting materials, such as green waste, animal manure, and food scraps, indicates the final product of the SOIL facilities is typical of compost in general and suggests the material has reached a safe endpoint for similar uses. See the Supporting Information for a more in-depth discussion of bacterial community changes throughout the composting process.

### Composting facility differences

While this study was not specifically aimed at comparing the two composting facilities, the differences in pile construction and maintenance likely contributed to differences observed in the degree of organism reduction during the thermophilic stage at Cap-Haitien compared to Port-au-Prince. The odor observed from Cap-H piles was most likely ammonia, which while not measured directly in this study, was apparently greater when working with the Cap-H samples. Finstein et al. [69] noted ammonia can have a disinfecting effect on compost. Because the piles at Cap-H are constructed on concrete slabs and the leachate is collect and reintroduced to the piles, it is very likely the nitrogen cycling dynamics are different at Cap-H than at PauP, leading to greater ammonia production and hence greater reductions of bacteria at the thermophilic stage.

## Conclusions

The present study documents the transformation of fecal-associated bacterial communities to ones recognizable as typical compost communities after thermophilic composting. PhyloChip analysis was used to track microbial community composition throughout the composting process, starting with identifying the predominantly human fecal bacterial populations and observing the substantial reduction of those taxa. The end-stage compost communities were similar to those reported from various other composting processes involving green waste, sewage sludge, and/or animal waste. It is the combination of high temperatures, rate of ascent (or at least use of nutrients), length of time at thermophilic temperatures [37,71,74], as well as the length of time after the thermophilic stage [5,85] that yields the most stable compost. The compost produced by SOIL from human waste with the addition of sugarcane bagasse and/or peanut shells was substantially different from the starting material and comprised bacteria commonly reported from other compost studies, suggesting an overall robustness of thermophilic composting to generate a valuable, nutrient-rich product that, in this case, also can help infrastructure-challenged communities meet their sanitary waste needs.

## Supporting information

S1 Fig Example temperature profile of a thermophilic composting bin in Port au Prince, Haiti at a SOIL facility. (TIF)

S1 Table PhyloChip probe intensity values (averages) for OTU with the greatest changes during thermophilic composting of human waste at Cap-Haitien, Haiti. Bar lengths represent values ranging from 10 to 15,860 fluorescence intensity units.
(XLSX)

S2 Table PhyloChip probe intensity values (averages) for OTU with the greatest changes during thermophilic composting of human waste at Port-au-Prince, Haiti. Bar lengths represent values ranging from 10 to 15,860 fluorescence intensity units.
(XLSX)

S3 Table Human gut microbiome ‘Common Core’ taxa (sensu Qin et al, 2010) present as a percentage of samples tested in fecal samples or in Haitian bucket samples. The samples from Haiti did not always have OTU classified to the same species, so additional rows were added to the table to accommodate the closest matches in the PhyloChip taxonomy. Grey/white shading demarcates taxa within the same family. Bar length represents the percentage of fecal samples (Qin et al., 2010) or Bucket samples containing OTU within a 'common core' taxon.
(XLSX)

S4 Table *Enterococcus* OTU probe intensity scores (indicating relative abundances) and differences between compost stages at each location. Values for each stage are average probe intensities for named species-level classifications or grouped as unclassified at the species level. Last three columns contain differences calculated between compost stages; negative values indicate decreases, whereas positive values potentially indicate regrowth. Kruskal-Wallis tests for differences in relative abundance among stages and post-hoc (Dunn) tests with BH corrected p-values are given below (bold values are significant at p<0.05).
(XLSX)

S5 Table ANOSIM (Analysis of Similarities) results for tests of differences between compost stages (one-way, ordered). Comparisons are based on Bray-Curtis similarity matrices of scaled, standardized, square-root transformed OTU intensities. Similarity percentages (SIMPER) routine-calculated average dissimilarities between groups are also included.
(XLSX)

S6 Table Cap-Haitien SIMPER (Similarity Percentages—Taxa contributions) ordered by decreasing Sim/SD (similarity within a group/standard deviation) or Diss/SD (dissimilarity between groups/standard deviation) to display the taxa contributing the most to similarity within a group or dissimilarity between groups (to a cumulative 5%). OTU intensities (reflecting relative abundances) were scaled, standardized, and then square-root transformed.
(XLSX)

S7 Table Port au Prince SIMPER (Similarity Percentages—Taxa contributions) ordered by decreasing Sim/SD (similarity within a group/standard deviation) or Diss/SD (dissimilarity between groups/standard deviation) to display the taxa contributing the most to similarity within a group or dissimilarity between groups (to a cumulative 5%). OTU intensities (reflecting relative abundances) were scaled, standardized, and then square-root transformed.
(XLSX)

S8 Table *E*. *coli* Testing using the MPN method at SOIL, Fall 2013—Spring 2014. These are the two piles (of 38 tested) that had high levels of E. coli at the beginning of the composting process. They were monitored throughout the composting process.
(XLSX)

S9 Table Phylum-level summed binary data were tested for differences across Stage at Cap-Haitien and Port au Prince SOIL thermophilic composting facilities. The alpha-level was adjusted to 0.0029 to account for multiple comparisons. Bold values in the Kruskal-Wallis (K-W) column indicate statistical significance. Posthoc comparisons among stages were performed using Dunn's method with BH correction; bold values indicate statistical significance. The alpha-level was adjusted to 0.0026 to account for multiple comparisons. Bold values in the Kruskal-Wallis (K-W) column indicate statistical significance. Posthoc comparisons among stages were performed using Dunn's method with BH correction; bold values indicate statistical significance.
(XLSX)

S10 Table Order-level summed probe intensities were tested for differences across Stage at Cap-Haitien and Port au Prince SOIL thermophilic composting facilities. The alpha-level was adjusted to 0.0026 to account for multiple comparisons. Bold values in the Kruskal-Wallis (K-W) column indicate statistical significance. Posthoc comparisons among stages were performed using Dunn's method with BH correction; bold values indicate statistical significance.
(XLSX)

S1 File Extended discussion of bacterial relative abundance changes throughout the thermophilic composting process. (PDF)
