# Supplementary figures and images for: Bacterial community structure transformed after thermophilically composting human waste in Haiti
Source: PLoS One. 2017 Jun 1;12(6):e0177626. doi: 10.1371/journal.pone.0177626 (PMC5453478; doi:10.1371/journal.pone.0177626)

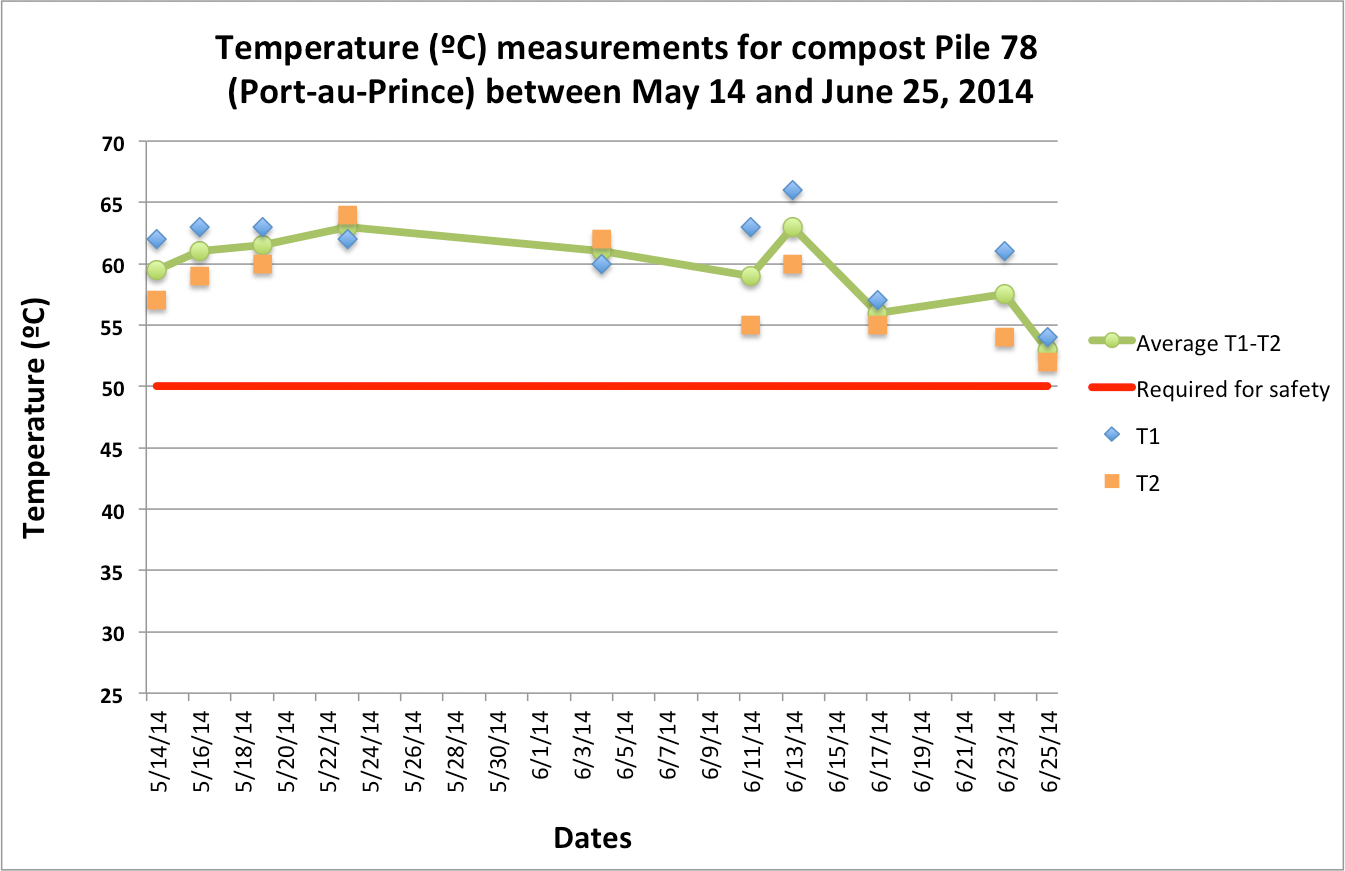

Supplement: S1 Fig — (TIF) [file pone.0177626.s001.tif]
