# Supplementary material for: Bacterial community structure transformed after thermophilically composting human waste in Haiti
Source: PLoS One. 2017 Jun 1;12(6):e0177626. doi: 10.1371/journal.pone.0177626 (PMC5453478; doi:10.1371/journal.pone.0177626)
Supplement: S1 File — (PDF) [file pone.0177626.s012.pdf]

Supplemental Information

Bacterial community structure transformed after thermophilically composting human waste in Haiti

Authors: Yvette M. Piceno[1] ¶, Gabrielle Pecora-Black[2] ¶, Sasha Kramer[3], Monika Roy[3], Francine C. Reid[1], Eric A. Dubinsky[1], Gary L. Andersen[1]*

[1]Ecology Department, Earth and Environmental Sciences Area, Lawrence Berkeley National Laboratory, Berkeley, CA, United States of America

[2] Agricultural & Environmental Chemistry Graduate Group, University of California, Davis, CA, United States of America

[3] Sustainable Organic Integrated Livelihoods, Port-au-Prince, Haiti

Extended Discussion

Microbial Community Dynamics

An interesting result of tracking changes throughout composting is illustrated by studies from two different groups: 1) Ishii et al. [1] and Ishii & Takii [2] noticed from lab and field studies of thermophilically-composted sewage sludge that similar groups of bacteria developed during the process; 2) Franke-Whittle et al. [3] reported similar end-stage communities developed despite starting with three different compost mixes 16 days earlier. Accordingly, we were interested in not only which bacteria declined during thermophilic composting, but also which increased.

The increases we observed for members of *Bacillus, Geobacillus,* and some of the Actinomycetales are consistent with other reports where these organisms were prominent in thermophilic composts [4]. Members of these groups have been shown to be able to degrade cellulosic and lignocellulosic materials [5,6]. *Bacillus*, *Geobacillus,* and other genera within the Firmicutes increased during the thermophilic stage and decreased afterwards. *Bacillus*, in particular, has been reported to dominate the thermophilic stage of composting regardless of the starting material, whether based on culture assays [7–10] or molecular assays [1,2,11,12], and *Bacillus* and *Geobacillus* were among the bacteria with the greatest increases in the present study. *Bacillus* spp. are known for producing proteases, amylases, and lipases [13,14]. *Geobacillus* has an array of enzymes for hydrolyzing plant matter [15], including lipids [16]. The substantial increase of these bacteria at the thermophilic stage is due to their heat tolerance (or preference) combined with their ability to degrade the readily available plant and fecal matter.

Other members of Class Bacilli that increased at the thermophilic stage were *Planifilum*, *Paenibacillus,* and *Ureibacillus. Planifilum* is classified within the Thermoactinomycetatceae. Yoshii et al. [10] recovered a *Planifilum* sequence by DGGE analysis of extremely thermophilic compost piles, reaching 90 – 100 °C at 50 cm below the surface. Strom [8], Ryckeboer et al. [17], and Rawat & Johri [4] also noted *Thermoactinomyces*, another genus within this family, has been recovered from various thermophilic and mesophilic composts, as was *Paenibacillus*. *Ureibacillus*

was dominant in a sample just cooling (at 45 ◦C) from the highest temperature point of a sewage sludge compost [12]. *Ureibacillus* can produce xylanase, ligninase, amylase, and cellulase (Ting et al., 2013) and have optimal growth temperatures of 50-60 ◦C [18], thus being well suited to thermophilic composting.

The delayed reduction of the Lactobacillales is consistent with other reports that showed *Lactobacillus* survived well through the thermophilic stage of mixed-animal zoo compost (collected on Day 60, temp. was 67 ◦C), as ~75% of the unassembled 16S rRNA gene sequences from metagenome data were affiliated with this genus [19]. The authors proposed the bacteria may have been involved with protein decomposition and carbon cycling.
In the current study, some *Lactobacillus* decreased markedly at the thermophilic stage (e.g., *L. ruminis*), while others remained fairly steady. Previous studies also show two kinds of responses for various members of the Lactobacillaceae: reports from Haruta et al. [20] and Peters et al. [11] showed greater abundances at mesophilic or early thermophilic stages, whereas Ishii & Takii [2] found them abundant in the thermophilic stage. Partanen et al. [14] reported *Lactobacillus* often accounted for a large proportion of 16S rRNA tag sequences from either feed or the 'feeding end' of compost drums where the temperature varied from 0 – 55 C. Thus, it appears temperatures in the lower range of the thermophilic spectrum do not restrict growth of some members of the Lactobacillaceae, at least in compost.

In the current study, many OTU within the Clostridiales decreased at least slightly at the Thermo stage, and this was likely due to the number of them originating from the fecal material and so unlikely to grow when the compost piles reached temperatures above 50 ◦C [21]. The main – and striking - exception within the Clostridiales was *Symbiobacterium thermophilum*, which increased dramatically at the thermophilic stage at both locations. This species was first established in co-culture with thermophilic *Bacillus* from compost [22]. Ueda and colleagues [23] suggested that while it was co-cultured with a thermophilic *Bacillus*, it is likely commensal with various bacteria in nature, and they documented its wide distribution (compost, soil, feces of numerous animals, feed). *S. thermophilum* produces tryptophanase and tyrosine-phenyl lyase [23], and genome analysis indicates it produces endospores [24]. Additionally, there were representatives of the genus *Clostridium* that did not decline at the Thermo stage at PauP. In two zoo compost metagenome samples, members of the Clostridiales (specifically *Clostridia*) had somewhat greater abundances than either *Bacillus* or *Geobacillus* (~5 – 15 % Clostridiales vs. ~2-9% Bacillales) [19]. Martins et al. suggest the Clostridiales are likely degrading cellulose and hemicellulose in the zoo compost. The SIMPER analysis also revealed OTU within *Desulfotomaculum* had elevated relative abundance in Thermo samples compared with Bucket samples., indicating a role for sulfur cycling at this stage.

Many members of the Actinobacteria increased at the thermophilic stage (*Thermobifida fusca, Saccharomonospora, Streptomonospora, Streptosporangium, Actinomadura*, and some Cellulomonadaceae and Micromonosporaceae) and did so to a greater degree at PauP than at Cap-H, though the increases were more consistently indicative of the Thermo stage at Cap-H according to the SIMPER results. Most then increased through to the Bagged stage at Cap-H, while peaking in the Curing stage at PauP. Several of the genera within Actinomycetales that increased in the thermophilic stage have been observed by others to be present in thermophilic compost (defined as spanning 45 - 70 ◦C), as reviewed by Ryckeboer et al. [17], Rawat & Johri [4], and discussed in Steger et al. [25]. *Thermobifida fusca* has an optimal growth temperature of 55 ◦C and has been found associated with both mesophilic and thermophilic compost stages previously [17,25]. The substantial and prolonged increase of *T. fusca*, among other Actinomycetes, throughout composting in the present study is consistent with *T. fucsa*

producing extraceulluar cellulases and lignocellulases important for plant cell wall degradation [19,26,27]. Specifically, *T. fusca* has been shown to produce lignocellulose-degrading enzymes when grown on sugarcane bagasse [28]. Trigo and Ball [26] also reported cellulolytic activity in *Saccharomonospora viridis* and many species of *Streptomyces*. *Saccharomonospora* has been reported from the hottest compost period [17,25] and also from later periods [10,12]. It can hydrolyse starch and xylan in addition to other compounds [2]. *Cellulomonas* within Cellulomonadaceae has been observed in the second mesophilic stage of compost [29] and various members of the family contain a suite of enzymes for carbohydrate metabolism as well as show chemotaxis toward cellobiose and hemicellulose degradation products [30].

In contrast to what was observed for most of the Actinobacteria, members of *Streptomyces, Actinomadura,* and especially *Arthrobacter* seemed to be sensitive to heat and so their greatest increases occurred later in the process, corroborated by the SIMPER results. *Streptomyces* has been recovered commonly from a variety of composts at both mesophilic and thermophilic stages [4,8,11,17] and was the dominant actinomycete cultured from sugarcane bagasse [31]. *Streptomyces* spp. are important not only because of their ability to degrade cellulose, chitin, xylan, and lignin [32,33], but also because of their extensive antibiotic production [33]. These traits combined make them great competitors for resources during composting. *Arthrobacter* has a more restricted appearance in composts. Ryckeboer et al. [17] listed *Arthrobacter* as being recovered only from mesophilic stages, Ishii et al. [1] observed it appearing in the late maturing phase of their study, and Steger et al. [25] reported *Arthrobacter* was the last of the actinomycetes to be detected, when the compost had cooled to a mean of 50 °C and then was dominant by Week 57, when the temperature had declined further to a mean of 45 °C. When considering the compost maturity index determined for the last experiment, the presence of *Arthrobacter* – and perhaps more so its presumed subsequent decline – may be a useful indicator of process completion. At 57 weeks, the compost analyzed by Steger *et al*. was considered 'curing' by the SOLVITA test, and it may be the Cap-H compost was of a similar stage when bagged, as *Arthrobacter* populations had increased in Bagged samples relative to the Curing samples, suggesting a longer maturation phase may be beneficial. By comparison, the PauP *Arthrobacter* population(s) appeared to have peaked in the Curing stage, so it may have been a more mature product. Jayasree & Balan [13] note *Arthrobacter* is prominent in soils and so also suggest their presence could be used as a marker for compost maturity. Other bacteria commonly associated with soil (Gemmatimonadetes, Planctomycetes, and Rhizobiales) also increased in the later stages, bolstering the argument of compost maturity.

The notable declines for the Bacteroidales and Enterobacteriales, especially steep at Cap-H, are likely for the same reasons as for the many Clostridiales. Reddy and colleagues [34] also noted decreased Bacteroidetes pyrotag sequences in compost-derived rice-straw incubations at thermophilic relative to mesophilic incubation temperatures. Regarding the Flavobacteriaceae, Danon et al. [35] found *Chryseobacterium* and *Flavobacterium* in their sewage sludge/yard waste compost most abundant around Day 41 (temperature around 30 °C) and tapering off but still prominent through Day 205. Our observation of members of the Class Sphingobacteria increasing at the Curing stage is consistent with observations by others [1,17], as was the increase of Planctomycetaceae [3,35]. *Dyella*, a member of the Xanthomonadaceae, likely increased in relative abundance in the Curing and/or Bagged stages because, similar to *Sphingomonas*, *Burkholderi*a, and some *Bacillus* and *Paenibacillus*, it is mesophilic and has the ability to degrade lignin [36]. Interestingly, *Halomonas* increased at both locations in the Curing phase. This may indicate a transient increase in salt concentration (osmotic pressure), as observed by Haruta et al. [20] with a food-based compost, though in that system, it was *Corynebacterium* and *Staphylococcus* that appeared prominently during this phase. Finally, we

observed various methyl-utilizing bacteria at their greatest relative abundance in the Curing or Bagged samples. *Methylocaldum* spp. are thermotolerant or thermophilic and have been isolated from compost previously [37]. Bacteria capable of using one-carbon compounds and that are mesophilic, such as *Methyloversatilis* [38], could be expected to do well after the thermophilic stage – as was observed in the current study - given the ongoing degradation of plant material as the piles cool.

References

1. Ishii K, Fukui M, Takii S. Microbial succession during a composting process as evaluated by denaturing gradient gel electrophoresis analysis. J Appl Microbiol. 2000;89: 768–777. doi:jam1177 [pii]
2. Ishii K, Takii S. Comparison of microbial communities in four different composting processes as evaluated by denaturing gradient gel electrophoresis analysis. JApplMicrobiol. 2003;95: 109–119.
3. Franke-Whittle IH, Knapp BA, Fuchs J, Kaufmann R, Insam H. Application of COMPOCHIP microarray to investigate the bacterial communities of different composts. Microb Ecol. 2009;57: 510–521. doi:10.1007/s00248-008-9435-2
4. Rawat S, Johri BN. Role of thermophilic microflora in composting. In: Satyanarayana T, Littlechild J, Kawarabayasi Y, editors. Thermophilic microbes in environmental and industrial biotechnology: biotechnology of thermophiles. Second. Dordrecht: Springer; 2013. pp. 137–169.
5. Rastogi G, Bhalla A, Adhikari A, Bischoff KM, Hughes SR, Christopher LP, et al. Characterization of thermostable cellulases produced by Bacillus and Geobacillus strains. Bioresour Technol. Elsevier Ltd; 2010;101: 8798–8806. doi:10.1016/j.biortech.2010.06.001
6. Pocas-Fonseca MJ, Matos RW de M, Mello-de-Sousa M. The lignocellulolytic system of thermophilic fungi and actinomycetes: structure, regulation, and biotechnological applications. In: Satyanarayana T, Littlechild J, Kawarabayasi Y, editors. Thermophilic microbes in environmental and industrial biotechnology: biotechnology of thermophiles. Second. Dordrecht: Springer; 2013. pp. 731–769.
7. Strom PF. Effect of temperature on bacterial species diversity in thermophilic solid waste composting. Appl Environ Microbiol. 1985;50: 899–905.
8. Strom PF. Identification of thermophilic bacteria in solid-waste composting. Appl Environ Microbiol. 1985;50: 906–913.
9. Blanc M, Marilley L, Beffa T, Aragno M. Thermophilic bacterial communities in hot composts as revealed by most probable number counts and molecular (16S rDNA) methods. FEMS Microbiol Ecol. 1999;28: 141–149. doi:10.1016/S0168-6496(98)00099-3
10. Yoshii T, Moriya T, Oshima T. Bacterial and biochemical properties of newly invented aerobic, high-temperature compost. In: Satyanarayana T, Littlechild J, Kawarabayasi Y, editors. Thermophilic microbes in environmental and industrial biotechnology: biotechnology of thermophiles. Second. Dordrecht: Springer; 2013. pp. 119–135.
11. Peters S, Koschinsky S, Schwieger F, Tebbe C. Succession of microbial communities during hot composting as detected by PCR-single- strand-conformation-polymorphism-based genetic profiles of Small-subunit rRNA genes. Appl Environ Microbiol. 2000;66: 930–936. doi:10.1128/AEM.66.3.930-936.2000
12. Su J-Q, Wei B, Ou-Yang W-Y, Huang F-Y, Zhao Y, Xu H-J, et al. Antibiotic Resistome and Its Association with Bacterial Communities during Sewage Sludge Composting. Environ Sci Technol. 2015;49: 7356–7363. doi:10.1021/acs.est.5b01012
13. Jayasree S, Balan R. Characterization and bacterial succession studies of garden waste

during the windrow composting process at Mercy College campus. Biochem Cell Arch. 2012;12: 403–413.
14. Partanen P, Hultman J, Paulin L, Auvinen P, Romantschuk M. Bacterial diversity at different stages of the composting process. BMC Microbiol. 2010;10: 94. doi:10.1186/1471-2180-10-94
15. Zeigler DR. The Geobacillus paradox: Why is a thermophilic bacterial genus so prevalent on a mesophilic planet? Microbiol (United Kingdom). 2014;160: 1–11. doi:10.1099/mic.0.071696-0
16. Charbonneau DM, Meddeb-Mouelhi F, Boissinot M, Sirois M, Beauregard M. Identification of Thermophilic Bacterial Strains Producing Thermotolerant Hydrolytic Enzymes from Manure Compost. Indian J Microbiol. 2012;52: 41–47. doi:10.1007/s12088-011-0156-8
17. Ryckeboer J, Mergaert J, Vaes K, Klammer S, Clercq D, Coosemans J, et al. A survey of bacteria and fungi occurring during composting and self-heating processes. Ann Microbiol. 2003;53: 349–410.
18. Weon HY, Lee SY, Kim BY, Noh HJ, Schumann P, Kim JS, et al. Ureibacillus composti sp. nov. and Ureibacillus thermophilus sp. nov., isolated from livestock-manure composts. Int J Syst Evol Microbiol. 2007;57: 2908–2911. doi:10.1099/ijs.0.65232-0
19. Martins LF, Antunes LP, Pascon RC, de Oliveira JCF, Digiampietri LA, Barbosa D, et al. Metagenomic Analysis of a Tropical Composting Operation at the São Paulo Zoo Park Reveals Diversity of Biomass Degradation Functions and Organisms. PLoS One. 2013;8: e61928. doi:10.1371/journal.pone.0061928
20. Haruta S, Kondo M, Nakamura K, Chanchitpricha C, Aiba H, Ishii M, et al. Succession of a microbial community during stable operation of a semi-continuous garbage-decomposing system. J Biosci Bioeng. 2004;98: 20–27. doi:http://dx.doi.org/10.1016/S1389-1723(04)70237-6
21. Wiegel J, Tanner R, Rainey FA. An introduction to the Family Clostridiaceae. In: Dworkin M, Falkow S, Rosenberg E, Schleifer K-H, Stackebrandt E, editors. The Prokaryotes Vol 4. Springer; 2006. pp. 654–678.
22. Ohno M, Shiratori H, Park MJ, Saitoh Y, Kumon Y, Yamashita N, et al. Symbiobacterium thermophilum gen. nov., sp. nov., a symbiotic thermophile that depends on co-culture with a Bacillus strain for growth. Int J Syst Evol Microbiol. 2000;50: 1829–1832. doi:10.1099/00207713-50-5-1829
23. Ueda K, Ohno M, Yamamoto K, Nara H, Mori Y, Shimada M, et al. Distribution and Diversity of Symbiotic Thermophiles,. Microbiology. 2001;67: 3779–3784. doi:10.1128/AEM.67.9.3779
24. Ueda K, Yamashita A, Ishikawa J, Shamada M, Watsuji TO, Morimura K, et al. Genome sequence of Symbiobacterium thermophilum, an uncultivable bacterium that depends on microbial commensalism. Nucleic Acids Res. 2004;32: 4937–4944. doi:10.1093/nar/gkh830
25. Steger K, Sjogren AM, Jarvis A, Jansson JK, Sundh I. Development of compost maturity and Actinobacteria populations during full-scale composting of organic household waste. J Appl Microbiol. 2007;103: 487–498. doi:10.1111/j.1365-2672.2006.03271.x
26. Trigo C, Ball AS. Production of Extracellular Enzymes During the Solubilization of Straw By Thermomonospora-Fusca Bd25. Appl Microbiol Biotechnol. 1994;41: 366–372. Available: <Go to ISI>://WOS:A1994NP65100015
27. Gomez Del Pulgar EM, Saadeddin A. The cellulolytic system of Thermobifida fusca. Crit Rev Microbiol. 2013;7828: 1–12. doi:10.3109/1040841X.2013.776512
28. Chen CY, Hsieh ZS, Cheepudom J, Yang CH, Meng M. A 24.7-kDa copper-containing oxidase, secreted by Thermobifida fusca, significantly increasing the xylanase/cellulase-

catalyzed hydrolysis of sugarcane bagasse. Appl Microbiol Biotechnol. 2013;97: 8977–8986. doi:10.1007/s00253-013-4727-y
29. Ryckeboer J, Mergaert J, Coosemans J, Deprins K, Swings J. Microbiological aspects of biowaste during composting in a monitored compost bin. J Appl Microbiol. 2003;94: 127–137. doi:10.1046/j.1365-2672.2003.01800.x
30. Stackebrandt E, Schumann P, Prauser H. The Family Cellulomonadaceae. The Prokaryotes: Actinobacteria. 2006;3: 983–1001. doi:10.1007/0-387-30743-5_40
31. Silva CF, Azevedo RS, Braga C, da Silva R, Dias ES, Schwan RF. Microbial diversity in a bagasse-based compost prepared for the production of Agaricus brasiliensis. Brazilian J Microbiol [publication Brazilian Soc Microbiol. 2009;40: 590–600. doi:10.1590/S1517-838220090003000023
32. Ramachandra M, Crawford DL, Hertel G. Characterization of an Extracellular Lignin Peroxidase of the Lignocellulytic Actinomycete Streptomyces viridosporus. Appl Environ Microbiol. 1988;54: 3057–3063.
33. Barka EA, Vatsa P, Sanchez L, Gaveau-vaillant N, Jacquard C, Klenk H-P, et al. Taxonomy, Physiology, and Natural Products of Actinobacteria. Microbiol Mol Biol Rev. 2016;80: 1–43. doi:10.1128/MMBR.00019-15.Address
34. Reddy AP, Simmons CW, D'haeseleer P, Khudyakov J, Burd H, Hadi M, et al. Discovery of Microorganisms and Enzymes Involved in High-Solids Decomposition of Rice Straw Using Metagenomic Analyses. PLoS One. 2013;8: e77985. doi:10.1371/journal.pone.0077985
35. Danon M, Franke-Whittle IH, Insam H, Chen Y, Hadar Y. Molecular analysis of bacterial community succession during prolonged compost curing. FEMS Microbiol Ecol. 2008;65: 133–144. doi:10.1111/j.1574-6941.2008.00506.x
36. Bugg TDH, Ahmad M, Hardiman EM, Singh R. The emerging role for bacteria in lignin degradation and bio-product formation. Curr Opin Biotechnol. 2011;22: 394–400. doi:10.1016/j.copbio.2010.10.009
37. Jäckel U, Thummes K, Kämpfer P. Thermophilic methane production and oxidation in compost. FEMS Microbiol Ecol. 2005;52: 175–184. doi:10.1016/j.femsec.2004.11.003
38. Kalyuzhnaya MG, De Marco P, Bowerman S, Pacheco CC, Lara JC, Lidstrom ME, et al. Methyloversatilis universalis gen. nov., sp. nov., a novel taxon within the Betaproteobacteria represented by three methylotrophic isolates. Int J Syst Evol Microbiol. 2006;56: 2517–2522. doi:10.1099/ijs.0.64422-0
